# Mating-Type System Analysis and Domestication of *Paramarasmius mesosporus* Based on Whole-Genome Sequencing

**DOI:** 10.3390/jof12080579

**Published:** 2026-08-05

**Authors:** Peng Zhu, Junling Wang, Jinjie Du, Shuainan Yang, Boran Zhang, Shuang Gao, Xiao Li, Ao Ma, Zengchi Wang, Jinghua Tian, Ming Li, Guojie Li, Shoumian Li

**Affiliations:** 1 College of Horticulture, Hebei Agricultural University, Baoding 071001, China; 15395614390@163.com (P.Z.); lixiao@hebau.edu.cn (X.L.);; 2 College of Life Sciences, Hebei Agricultural University, Baoding 071001, China; 3 Center for Experimental and Practical Training, Hebei Agricultural University, Baoding 071001, China; 4 Department of Agriculture and Animal Husbandry Engineering, Cangzhou Technical College, Cangzhou 061001, China

**Keywords:** *Paramarasmius mesosporus*, tetrapolar mating system, artificial domestication, Hi-C, chromosome-level genome, MAPK pathway, sexual reproduction, germplasm innovation

## Abstract

*Paramarasmius mesosporus* is a wild edible mushroom with a strong aroma and high culinary value, capable of growing at temperatures up to 36 °C. However, its domestication system and the genetic basis of its sexual reproduction remain largely unexplored. In this study, fresh fruiting bodies of *P. mesosporus* were collected from the rhizosphere of *Imperata* spp. in saline–alkali land in Hebei Province, China. The species was successfully domesticated for the first time, and fruiting bodies were harvested 7–10 days after spawning. A chromosome-level genome of the monokaryotic strain ‘Q2’ was assembled using whole-genome sequencing, transcriptome analysis, and Hi-C technology, with a total size of 46.57 Mb that was anchored onto 11 pseudochromosomes at a mounting rate of 95.18%. The mating-type system was identified as a typical tetrapolar heterothallic type, with the MAT A and MAT B loci located on chromosomes 2 and 10, respectively. The MAT A locus encodes *HD1*, *HD2*, *MIP*, and *β-fg*, among which *tr-HD1* was found to be pseudogenized due to domain truncation. The MAT B locus comprises seven pheromone receptor genes and seven pheromone precursor genes arranged in an interspersed pattern. Based on these genomic features, a preliminary MAPK signaling pathway model was proposed to elucidate the molecular regulation of sexual reproduction. This study achieved the first artificial domestication and cultivation of *P. mesosporus*. systematically elucidated genomic characteristics and mating-type molecular mechanisms of this species were systematically elucidated, providing a theoretical foundation for hybrid breeding, molecular marker-assisted selection, and genetic improvement. These findings hold significant scientific and applied value for germplasm innovation and industrial development of this rare edible mushroom.

## 1. Introduction

*Paramarasmius mesosporus*, originally named as *Marasmiellus mesosporus*, was originally described by Vladimír Antonín in 2022. Then this species was identified as a member of the genus *Paramarasmius* based on morphological and molecular phylogenetic evidences. The main morphological characteristics of this species are as follows: fruiting bodies small-sized, solitary or gregarious; pileus 0.5–4 cm in diameter, round, with a wavy margin, and obvious radial striations on the lamellae [1]. Due to its habitat on the base of thatch grass, this species is colloquially named “Mao Wo-er” (thatch tussock mushroom) in the production region. Its wild fruiting bodies are characterized by a unique flavor and delicious taste, and have currently been brought into the marketization stage, demonstrating considerable development potential. Of particular importance, wild strains of *P. mesosporus* usually fruit under high-temperature conditions up to 35 °C during summer and in saline–alkali soil environments, showing excellent thermotolerance. The frequency of extreme high-temperature events has been significantly increased because of global climate warming [2,3], whereas most of the currently cultivated edible fungi are low- to medium-temperature species, and thermotolerant cultivars suitable for summer cultivation are found to be extremely scarce. Therefore, important strategic significance is attached to the exploration and domestication of new thermotolerant wild edible mushroom resources for ensuring the summer supply of the edible mushroom industry and addressing climate change.

Edible fungi are not only highly valued for their rich nutritional composition but also possess significant potential in biological control [4,5]. China has become the world’s largest producer and consumer of edible fungi [6]. The exploration and domestication of wild edible mushroom resources have been recognized as an important pathway for deepening the sustainable utilization of fungal resources as market demand becomes increasingly diversified [7,8,9]. However, a low fruiting body yield remains in the initial domestication practice of *P. mesosporus*, and its industrial development is severely limited by this issue. The development of new high-yield strains through hybrid breeding has been shown by modern edible mushroom breeding to be a key approach to overcoming the yield bottleneck. The theoretical foundation for crossbreeding is provided by the elucidation of the mating-type system. Sexual reproduction in basidiomycetes is jointly regulated by two unlinked mating-type loci, A (MAT A) and B (MAT B), which determine the pairing affinity between strains and the formation of dikaryotic mycelia with fruiting ability [10,11]. Homeodomain transcription factors (*HD1*/*HD2*) are encoded by the MAT A loci, and nuclear pairing and clamp connection formation are mainly regulated by them. Pheromone precursors and their receptors are encoded by the MAT B loci, and the regulation of nuclear migration and hyphal fusion is accomplished by them [12,13]. Intraspecific compatibility is determined not only by the allelic diversity and structural characteristics of mating-type loci, but also the evolutionary history and population genetic structure of the species are reflected by them [14]. Therefore, important guidance for understanding the mating system of newly domesticated species and for formulating scientific crossbreeding strategies is provided by systematic dissection of the genetic structure of mating-type loci.

During the sexual reproduction process of basidiomycetes, the MAPK signaling pathway serves as the central hub for pheromone recognition and signal transduction. When monokaryotic hyphae carrying different mating-type alleles are brought into contact, mutual recognition between the pheromones and their receptors encoded by the B locus is achieved, and the downstream MAPK cascade is activated. Key events such as cell fusion and nuclear migration are subsequently induced by this cascade, ultimately leading to the formation of dikaryotic hyphae with fruiting ability [15,16]. In addition, mating efficiency can be regulated by environmental factors such as light and temperature through crosstalk with other signaling pathways [17]. Therefore, systematic dissection of the mating-type locus structure and the MAPK signaling pathway is considered an important prerequisite for revealing the molecular mechanism of sexual reproduction in basidiomycetes and for guiding crossbreeding practice.

To date, no systematic studies have been reported on the artificial domestication, genetic background, or mating system of *P. mesosporus*. The chromosome-level genome and the molecular characteristics of its mating-type loci remain completely unknown. Basic biological research and genetic improvement applications of this species are severely restricted by this situation. To narrow these research gaps, artificial domestication of *P. mesosporus* was carried out for the first time. A chromosome-level genome map of *P. mesosporus* was constructed using whole-genome, transcriptome, and Hi-C sequencing. MAT A and MAT B structures were dissected, a tetrapolar heterothallic mating system was confirmed, and a MAPK model was built. Theoretical basis for crossbreeding and marker-assisted selection, and molecular foundation for sexual reproduction regulation, are provided.

## 2. Materials and Methods

### 2.1. Tested Strain

Wild samples of *Paramarasmius mesosporus* were collected from the living roots of thatch grass (*Imperata* spp.) growing on saline–alkali land in Yanshan County, Cangzhou City, Hebei Province. The coordinates are 38.25° N, 117.77° E. A strain of *P. mesosporus* was obtained using the fruiting body tissue isolation method. The strain was derived from asexual tissue which was taken from the junction between the pileus and the stipe. Then it was preserved on potato dextrose agar (PDA) medium, which was prepared with 200 g/L potato infusion, 20 g/L glucose, and 20 g/L agar, topped up to 1 L with deionized water, and sterilized at 121 °C for 40 min. The culture was stored at 4 °C for later use.

### 2.2. Strain Identification

The wild fruiting bodies of *P. mesosporus* were observed and described for macroscopic and microscopic characteristics, including pileus, lamellae, stipe, clamp connections, and spore morphology. Genomic DNA was extracted from wild and cultivated fruiting bodies, as well as activated mycelial cultures of *P. mesosporus* using a modified cetyltrimethyl ammonium bromide (CTAB) method [18]. PCR amplification was performed using the forward primer ITS5 (5′-GGAAGTAAAAGTCGTAACAAGG-3′) and the reverse primer ITS4 (5′-TCCTCCGCTTATTGATATGC-3′) [19]. The amplified products were subjected to Sanger bidirectional sequencing by Beijing Biomed Gene Technology Co., Ltd. (Beijing, China). The obtained sequences were subjected to BLAST alignment against published reliable sequences in the GenBank database using the NCBI BLAST (version 2.17.0) server [20]. The ITS sequence of *Chaetocalathus liliputianus* was used as an outgroup. Multiple sequence alignment was performed using MEGA 12.0.11 [21]. The sequences were trimmed using the online tool Gblocks [22]. A phylogenetic tree was constructed using IQ-TREE 3.0.1 based on the maximum likelihood (ML) method [23].

### 2.3. Domestication

#### 2.3.1. Domestication Site

The domestication site was located in a small sunlight greenhouse at the Innovation Experimental Garden of the West Campus of Hebei Agricultural University, Lianchi District, Baoding City, Hebei Province, China. The coordinates are 38.49° N, 115.26° E.

#### 2.3.2. Preparation of Cultivated Spawn

The tested strain preserved on a PDA slant was transferred to a PDA plate for activation. The culture was incubated at a constant 25 °C. After activation, mycelial plugs (1 cm in diameter) were cut under sterile conditions using a punch. Three plugs were then inoculated into the prepared spawn culture medium (The substrate composition is expressed on a dry weight basis (89% cottonseed hulls, 10% wheat bran, and 1% gypsum) and that water was added to achieve a final moisture content of 60–65% (*w*/*w*).) was loaded into polypropylene bags (17 cm × 35 cm) at 800 g per bag and sterilized at 125 °C for 2 h. The culture was placed at 25 °C in the dark until the bag was fully colonized by the mycelia, which was then considered as spawn.

#### 2.3.3. Preparation of Fermented Substrate

The roots of the wheat straw were removed, and the remaining straw was rinsed with clean water for the removal of soil and impurities, then cut into approximately 10 cm segments. The straw segments were mixed thoroughly with dried corncobs (crushed to 5 cm) at a mass ratio of 1:1. The mixed materials were piled into a frustum-shaped fermentation stack with a base diameter of 150 cm in height and 200 cm in width. A thermometer was inserted into the core for real-time temperature monitoring of the material temperature. The surface was sprinkled with water daily to maintain humidity and promote softening promotion on a daily basis. The fermented substrate was maintained for 24 h, followed by turning when the stack temperature rose to 56 °C. The fermented substrate was stirred evenly and then replied. This turning operation was repeated twice. The pH of the fermentation material was adjusted to 8.0–8.5 with quicklime, and the water content was set to 58–60%.

#### 2.3.4. Domestication and Fruiting

The spawn was broken into pieces of 1.5–2.0 cm in diameter. A 10 cm-thick layer of fermentation material was then laid on the greenhouse floor. The inoculation amount was set at 0.5–0.6 kg/m^2^. The spawn pieces were evenly sown into the fermented substrate at a depth of 5–6 cm, with a spacing of 8–10 cm between pieces. Finally, the surface of the material was evenly covered with 1.5–2 cm of soil, followed by a 2–3 cm-thick layer of wheat straw that had been soaked in 1% lime water for 24 h. Air humidity was maintained at 85–90%, temperature was maintained at 28–35 °C, and water was sprayed appropriately to keep the covering soil moist. The greenhouse roof was covered with shade nets to reduce light and temperature. The optimal light intensity was determined to be 500–1000 lx. A photoperiod of 14 h light and 10 h dark was applied, and the CO_2_ concentration was maintained below 0.08% during the cultivation period. Harvesting was performed when the pileus was fully grown but before its margin began to curl outward. The pileus diameter and thickness, stipe length and diameter were measured, and the spore morphology was observed under a microscope (ECLIPSE Ni-U, Nikon Corporation, Tokyo, Japan). The total nitrogen content of the fruiting bodies was determined by the Kjeldahl method, and the crude protein content was calculated accordingly [24].

### 2.4. Chromosome-Level High-Quality Genome Fine Map

#### 2.4.1. Acquisition of Monokaryotic Strains and DNA/RNA Preparation

Spores of the fruiting body were collected following the method of Zhang et al. with minor modifications [25]. The pilei of completely mature fruiting bodies were excised then surface-sterilized by immersion in 75% (*v*/*v*) medical alcohol for 30 s. The sterilized pilei were then transferred into a new sterile 100 mL Erlenmeyer flask, into which 5 mL of sterile water was added. The flask was shaken gently to facilitate spore release, and the resulting spore suspension was collected for subsequent use. Next, the spores were diluted to 10^3^ spores/mL with a hemocytometer, spread on PDA medium, and incubated at 25 °C for 7 d. Germinated colonies were picked and subcultured. A sterilized coverslip was inserted into the medium at a 45 angle when the colonies were visible to the naked eye. The coverslip was removed after the hyphae had grown to approximately half the length of the coverslip. The sample was fixed with 4% RNase-free paraformaldehyde (Yuanye Biotechnology, Shanghai, China), washed with 1× PBS buffer (Solarbio, Beijing China) to remove the fixative, and then dehydrated through a graded ethanol series (25%, 50%, 75%, and 100%). Finally, the sample was mounted in anti-fade mounting medium containing DAPI (UElandy, Suzhou, China) and preserved at room temperature for 24 h. The fluorescent sections were observed under an upright fluorescence microscope (Nikon ECLIPSE Ci-L plus, Nikon Corporation, Tokyo, Japan). Hyphae lacking clamp connections were considered monokaryotic. After the monokaryotic strain ‘Q2’ was selected and incubated at 25 °C in the darkness for 5 d, the colony was cut into small pieces (2 mm × 2 mm × 1 mm) and ten pieces were inoculated into liquid medium (200 g potato, 20 g glucose, 2 g peptone, 1 g MgSO_4_, 1 L deionized water, sterilized at 121 °C for 40 min). After static culture for 12 h, the culture was incubated with shaking at 32 °C and 150 r/min for 5 d. Mycelia were collected and blotted dry with sterile filter paper, and genomic DNA was extracted using a modified CTAB method. On the other hand, total RNA was extracted using the EZNA kit (Omega, Stamford, CT, USA) for transcriptome analysis.

#### 2.4.2. Genome Sequencing and Assembly

The *P. mesosporus* strain ‘Q2’ was subjected to whole-genome CCS sequencing by Biomarker Technologies Co., Ltd. (Beijing, China) using the PacBio Sequel II platform. A total of 2.78 Gb of CCS reads were obtained. The CCS reads were then assembled using Hifiasm v0.16.1 [26]. The assembled genome was further corrected with second-generation sequencing data using Pilon software v1.22 [27]. Genome assembly completeness was evaluated using BUSCO v2.0 [28]. RNA quality was assessed using the RNA Nano 2000 assay kit (Thermo Scientific, Waltham, MA, USA) and the Qubit RNA assay kit on a Qubit 2.0 fluorometer (Life Technologies, Carlsbad, CA, USA). RNA samples were sequenced on the Illumina NovaSeq platform (Illumina, San Diego, CA, USA), and 7.57 Gb of clean reads were obtained for genome annotation.

#### 2.4.3. Construction of Hi-C Libraries and Chromosomal Assembly

DNA extraction, Hi-C library construction, sequencing, and assembly were carried out by Biomarker Technologies Co., Ltd. (Beijing, China). First, cells were fixed with formaldehyde to maintain the spatial conformation of DNA. After digestion with the restriction endonuclease Dpn II, the ends were repaired and labeled with biotin. Interacting fragments were ligated in dilute solutions to form circular products. Cross-linking was reversed, and DNA was sheared. Biotin-labeled fragments were captured using streptavidin-coated magnetic beads, and a Hi-C sequencing library was constructed. The library concentration and insert size were measured respectively by means of Qubit 2.0 and Agilent 2100. The effective concentration of the library was quantified by q-PCR to ensure quality. Sequencing was performed on the Illumina Hiseq platform with PE150 read length. Clean reads were aligned to the reference genome using BWA [29]. Valid and invalid interaction pairs were identified using HiC-Pro (v2.10) [30]. The genomic sequences were clustered, ordered, oriented, and assembled to the chromosome level using LACHESIS [31].

#### 2.4.4. Genome Annotation

Gene structure prediction was performed using ab initio prediction, homology-based prediction, and transcriptome evidence prediction, and the final gene set was obtained by consolidating the three predictions. Ab initio prediction was carried out using Genscan v1.0 [32], Augustus v2.4 [33], GlimmerHMM v3.0.4 [34], GeneID v1.4 [35], and SNAP (version 2006-07-28) [36]. Homology-based prediction was performed using GeMoMa v1.3.1 [37].

BLAST alignment was performed against the KOG, KEGG [38], Swiss-Prot [39], TrEMBL [39], and Nr [40] functional databases to obtain gene functional annotation results based on the predicted gene sequences. GO [41] functional annotation was performed using Blast2GO [42] based on the Nr database alignment results. Protein domain annotation was performed using HMMER [43] against the Pfam [44] database.

### 2.5. Mating-Type Gene Analysis

The fine gene structure maps of the mating-type genes were drawn using the IBS website [45], and gene structure visualization was performed using the GSDS website [46]. Finally, chromosomal localization maps, similarity comparison of the gene sequences at the mating-type B locus, and genome-wide synteny analysis were carried out using Tbtools [47].

The gene sequences and structures of the mating-type A and B loci were determined based on the genome annotation results of the monokaryotic strain ‘Q2’ and the NCBI-CDD database [48] for conserved domain prediction in target proteins (visualized with BioPlot at https://chiplot.online/index.html, accessed on 2 May 2026). HD genes were identified by phylogenetic analysis. The protein domains of HD genes were analyzed using InterproScan [49] and CDD. Nuclear localization signals of *HD1* and *HD2* were predicted by PSORT II [50]. The secondary and tertiary structures of HD proteins were predicted using SOPMA [51] and SWISS-MODEL [52].

The transmembrane structures of pheromone receptors were analyzed using TMHMM Server [53]. The identification of the pheromone receptor genes was followed by a search for open reading frames (ORFs) within 5 kb upstream and downstream of each gene. Short peptides with amino acid lengths ranging from 20 to 100 aa and containing a CaaX conserved motif at the 3′ end were then searched. The obtained conserved domains were analyzed using the KEGG database and CDD, and the pheromone receptor signaling response pathway of *P. mesosporus* was constructed based on the yeast MAPK signaling pathway.

## 3. Results

### 3.1. Morphological Identification and Phylogenetic Tree

**Figure 1:**
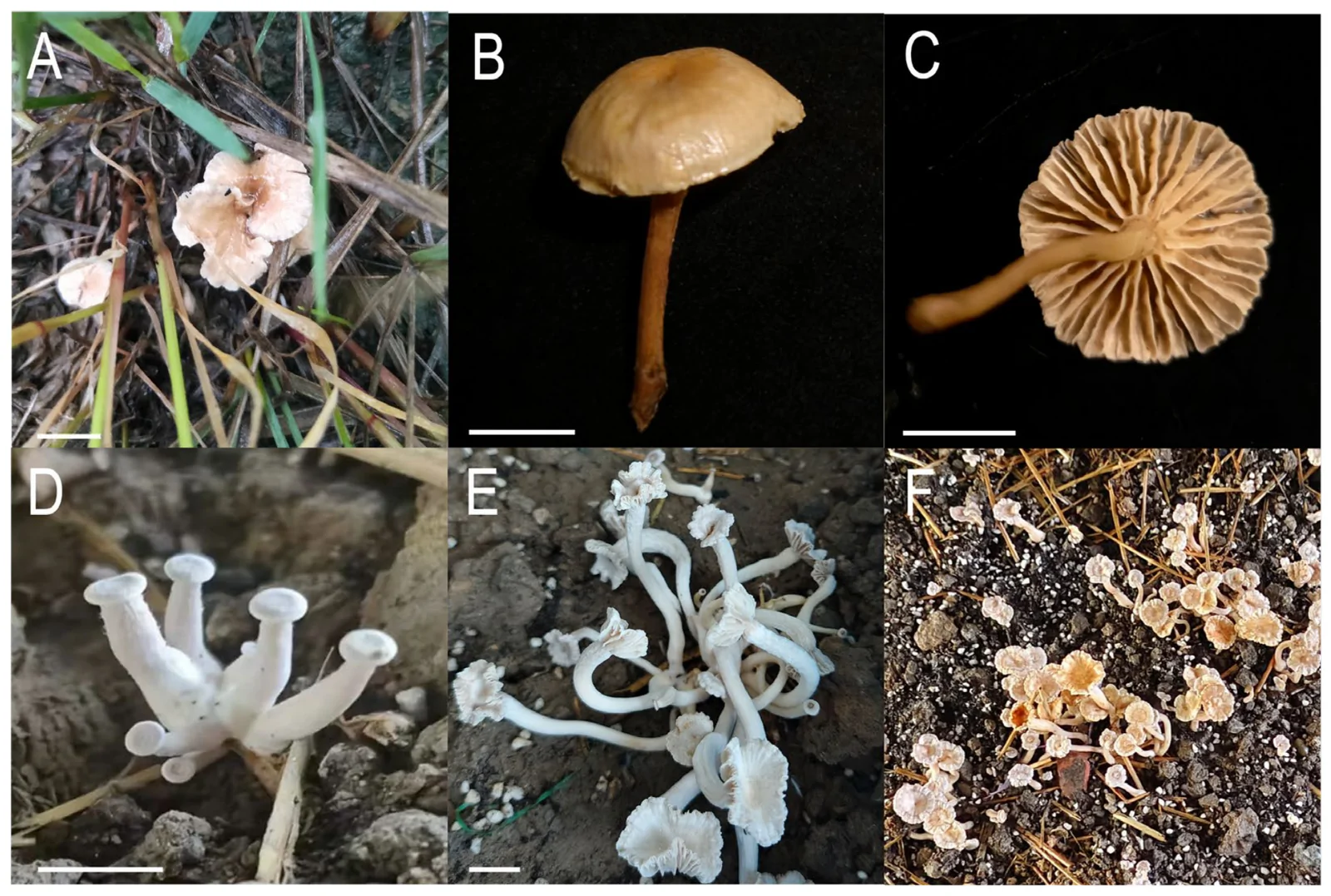
(**A**) Wild fruiting body. (**B**) Front view of the wild fruiting body. (**C**) Back view of the wild fruiting body. (**D**) Young cultivated fruiting body. (**E**) Mature cultivated fruiting body. (**F**) Fruiting bodies at the domestication site. Note: Scale bars = 1 cm.

The pileus of the wild fruiting body is flattened with a diameter of 1.0–4.0 cm upon maturity, as shown in Figure 1. The center is slightly depressed. The color is flesh white to grayish white. The surface is smooth or has fine villi. The lamellae are relatively sparse with abundant lamellulae, radially arranged, and are flesh white. The stipe is slender and cylindrical, 3.0–8.0 cm in length and 0.2–0.5 cm in diameter. The texture is flexible, and the surface is smooth. The pileus is light yellow, and the stipe is yellowish-brown in mature fruiting bodies. These morphological characteristics are consistent with those of *P. mesosporus* [1].

**Figure 2:**
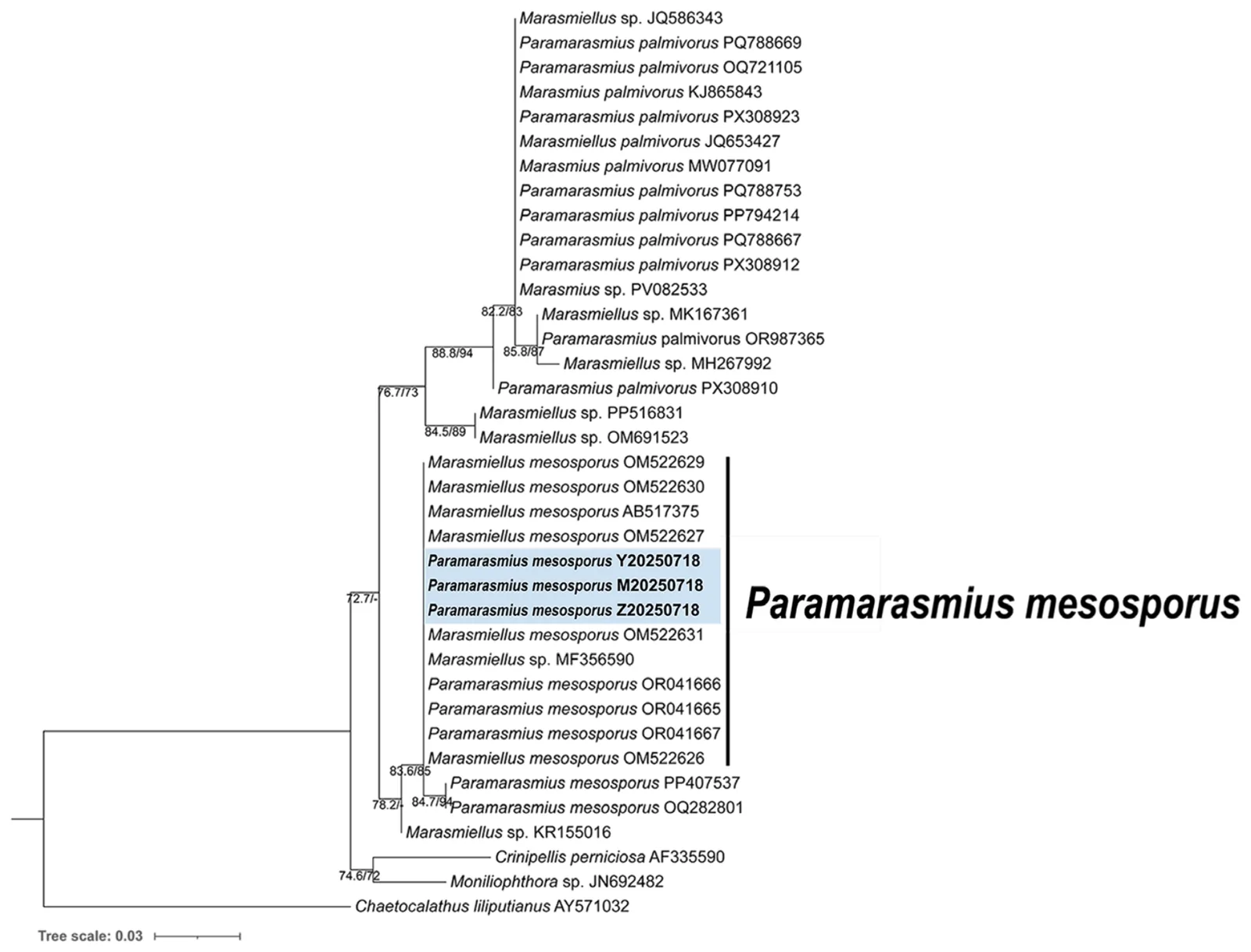
ITS phylogenetic tree of *P. mesosporus* and its closely related species constructed using the maximum likelihood (ML) method. Note: The wild fruiting body, cultivated fruiting body, and mycelium from the tissue-isolated strain are represented by Y20250718, Z2025071, and M20250718, respectively, and are located in the blue-shaded area. The SH-aLRT support values and UFBoot support values from the maximum likelihood analysis are indicated by the numbers below/before and after the branch nodes, respectively.

The ITS sequences of the wild fruiting bodies, cultivated fruiting bodies, and mycelia from the tissue-isolated strains were found to have 100% similarity and query coverage with *P. mesosporus* specimens OR041667, MF356590, and OM522626, as shown in Figure 2. These sequences were clustered together, and a highly supported clade was formed (with SH-aLRT support >83% and UFBoot support >85% in maximum likelihood analysis). The fruiting bodies and strains used in this study were identified as *P. mesosporus* based on morphological identification, sequence alignment, and phylogenetic analysis.

### 3.2. Domestication Results

**Figure 3:**
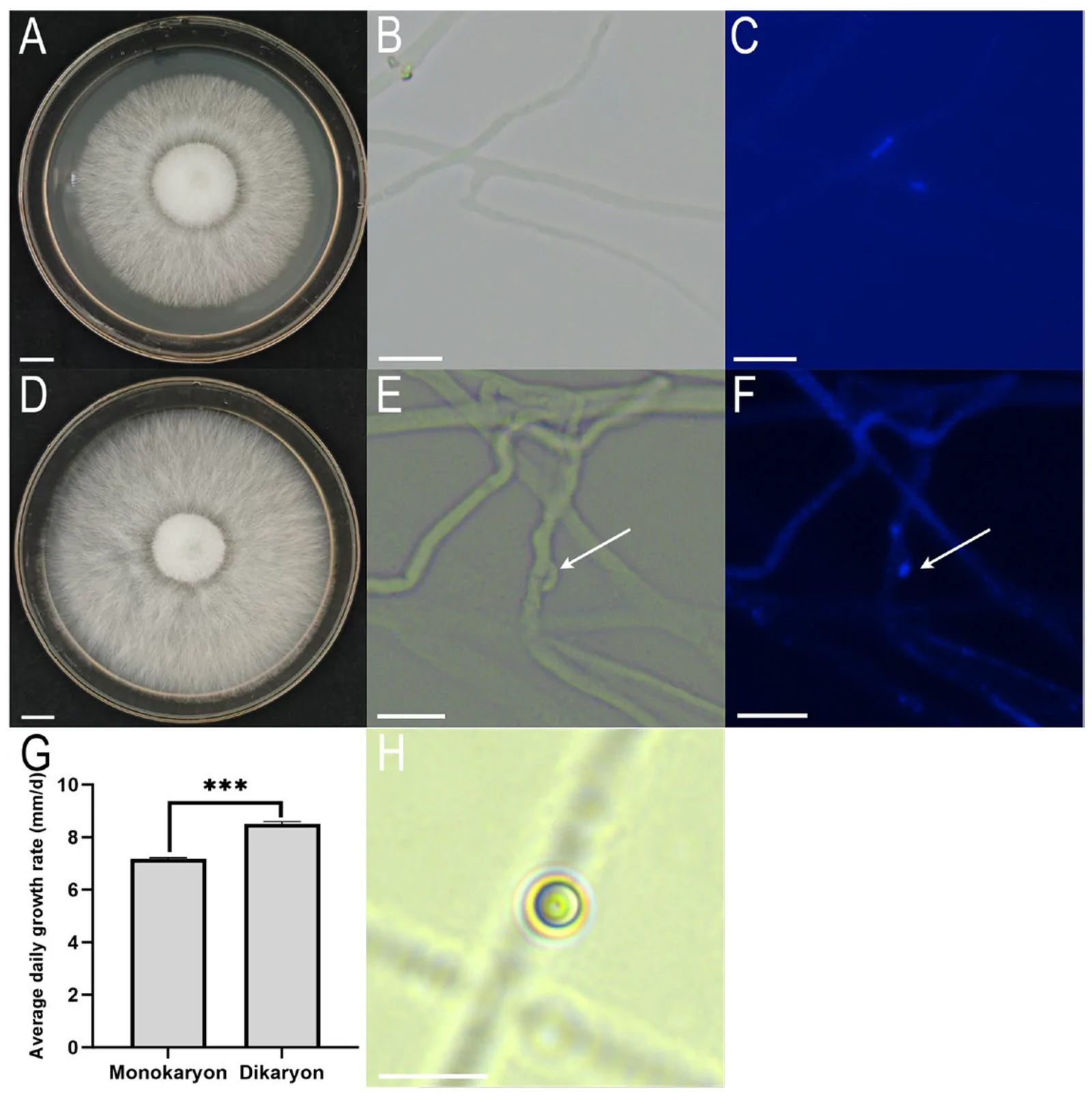
(**A**) Colony morphology of monokaryotic hyphae on PDA medium. (**B**) Bright-field micrograph of monokaryotic hyphae. (**C**) Fluorescence micrograph of monokaryotic hyphae. (**D**) Colony morphology of binucleate mycelium on PDA medium. (**E**) Colony morphology of dikaryotic hyphae on solid medium. (**F**) Fluorescence micrograph of dikaryotic hyphae. (**G**) Comparison of average daily growth rate (mm/d) between monokaryotic and dikaryotic hyphae. (**H**) Micrograph of spore morphology. Note: Scale bar: (**A**,**D**) = 1 cm, (**B**,**C**,**E**,**F**,**H**) = 10 μm. Arrows indicate forming clamp connections. *** = *p* < 0.001.

Primordia formed 3 d after sowing, and fruiting bodies matured 7 d later, as shown in Figure 1 The fruiting bodies were solitary or in clusters, with most fruiting bodies growing on wheat straw. The morphological characteristics of artificially cultivated *P. mesosporus* fruiting bodies are shown in Figure 1 The pileus of young fruiting bodies is round with a margin rolled outward, and the entire fruiting body is grayish white. In mature fruiting bodies, both the pileus and stipe are light yellow. The pileus margin rolls upward by over-harvesting. The pileus diameter is 1–3.5 cm, and the pileus thickness is 0.1–0.4 cm. The lamellae are sparse and short, with many lamellulae. The stipe is 7–13 cm long and 0.3–1.0 cm in diameter. The basidiospores are green, elliptical to nearly round, with a diameter of 2.4–5 µm (Figure 3). No distinctive odor is detected in the fruiting bodies. The crude protein content of the air-dried fruiting bodies is 7.52%.

### 3.3. Fine Genomic Map of P. mesosporus Q2

**Figure 4:**
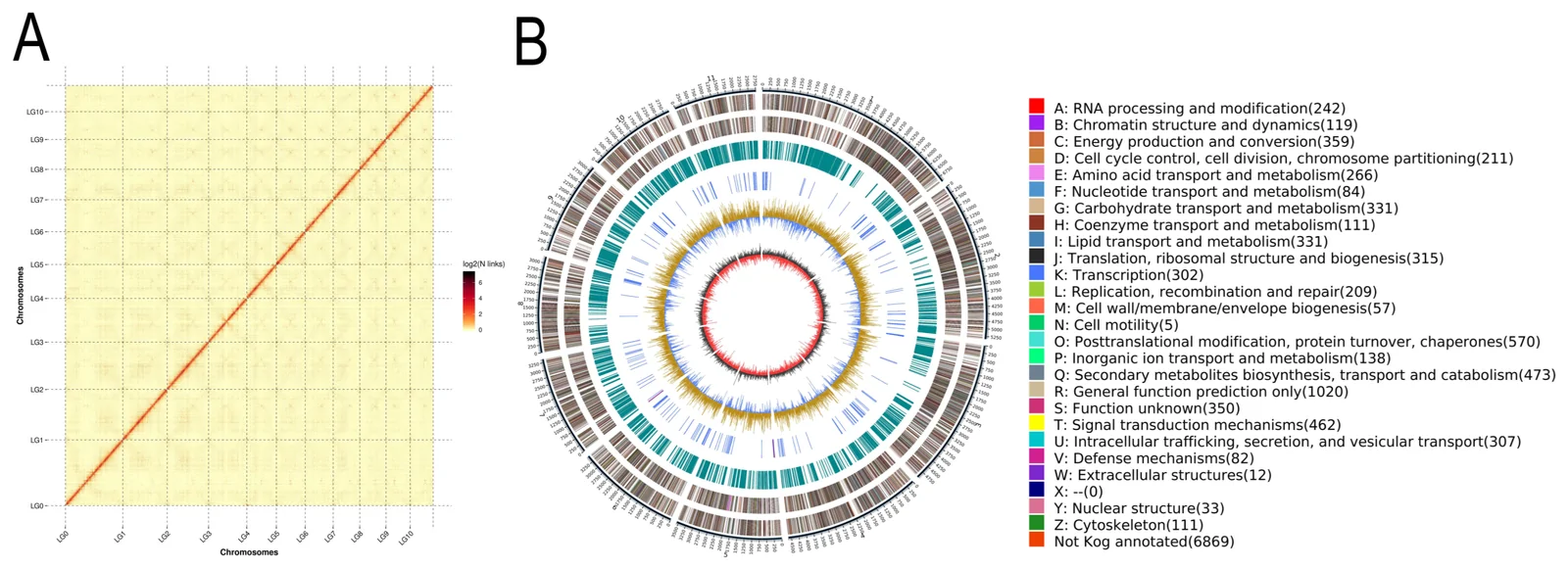
Genomic map of *P. mesosporus*. (**A**) Hi-C interaction heatmap of *P. mesosporus*. (**B**) Circular genome map of *P. mesosporus*. Note: Lachesis Groups 00 to 10 are represented by LG00 to LG10. The order of each bin on the corresponding chromosome group is indicated by the *X*-axis and *Y*-axis. The positional coordinates of chromosomes 1–11 are represented by the outermost circle. Gene density, repetitive sequence density, GC content, and transcriptome coverage are shown by the tracks from the outside to the inside.

Monokaryotic and dikaryotic strains, along with strain growth and microscopic imaging results, are shown in Figure 3, and the strain used for genome sequencing was confirmed to be monokaryotic. A fine genomic map of the monokaryotic strain ‘Q2’ was constructed by integrating genomics, transcriptomics, and Hi-C sequencing technologies, and its mating-type loci and MAPK signaling pathway were systematically analyzed, as shown in Figure 4. The complete genome size of *P. mesosporus* is 46.57 Mb. The contig N50 length is 3.48 Mb, the scaffold N50 length is 3.56 Mb, the sequencing depth is 59.58×, and the GC content is 46.16%. The genome completeness assessed by BUSCO is 95.17%. Chromosome-level genome assembly was achieved by Hi-C-assisted chromosome scaffolding. A total of 44.33 Mb of sequence was assigned to 11 chromosomes, with a mounting rate of 95.18% (Table A1). A total of 12,599 protein-coding genes were predicted in the whole genome. Among them, 12,373 genes were successfully annotated by BLAST alignment against the GO, KOG, and KEGG functional databases (Table A2). The first chromosome-level genome assembly of *P. mesosporus* is reported in this study.

### 3.4. Protein-Coding Gene Prediction

**Figure 5:**
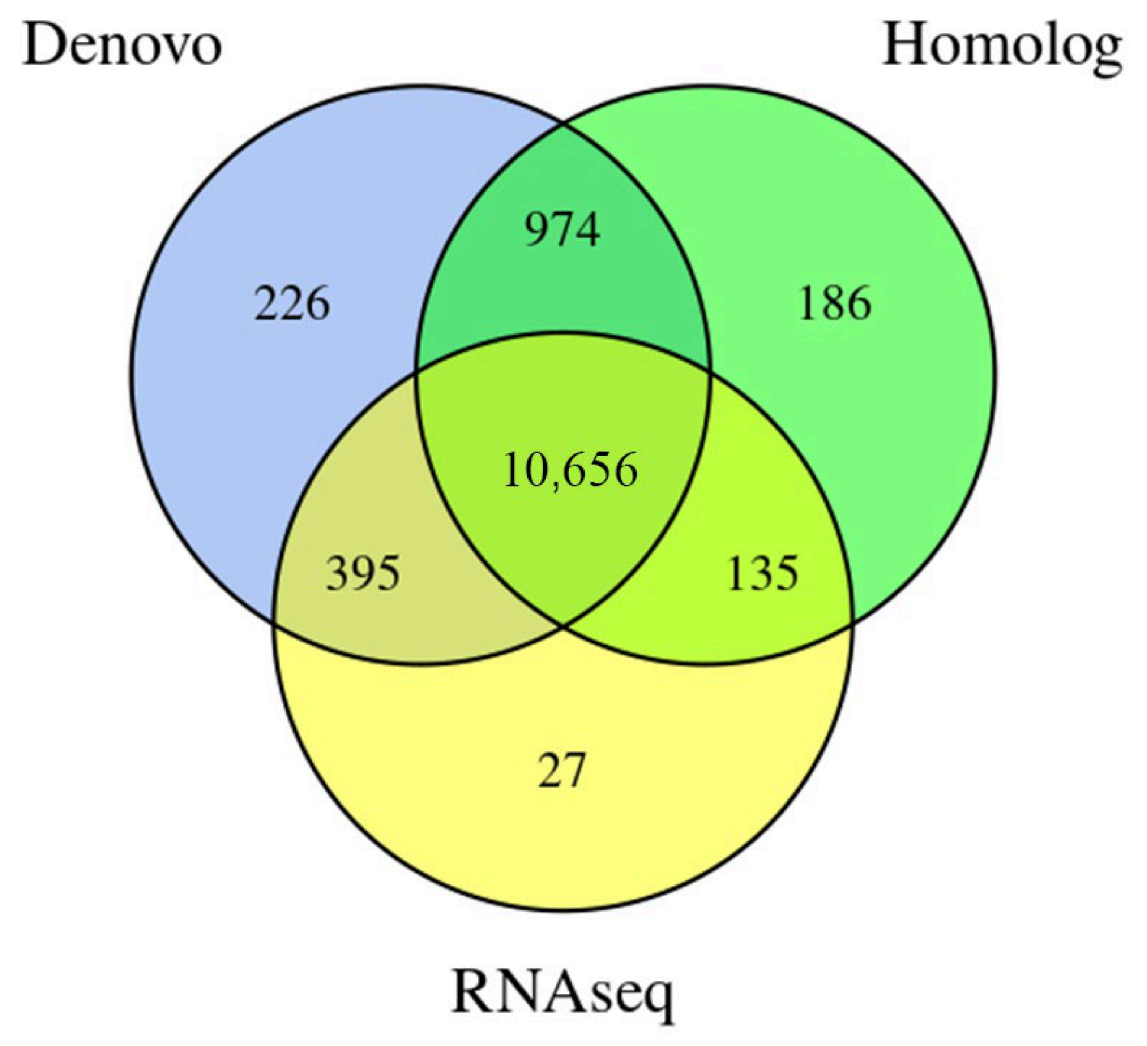
Statistical chart of three gene prediction strategies for *P. mesosporus*.

Three strategies were mainly used for gene structure prediction: ab initio prediction, homology-based protein alignment, and transcriptome evidence, as shown in Figure 5. Among the 12,599 predicted genes, 12,373 were supported by either homology-based prediction or transcriptome evidence, with a support rate of 98.2%. A total of 10,656 genes were simultaneously predicted by all three methods, accounting for 84.58% of the consistent predictions. Only 226 genes (1.79%) were predicted solely by ab initio methods, 27 genes (0.21%) were supported only by transcriptome evidence, and 186 genes (1.48%) were supported only by homology-based protein alignment. High reliability of the gene prediction results in this study was indicated by these results.

#### Genome Functional Annotation

**Figure 6:**
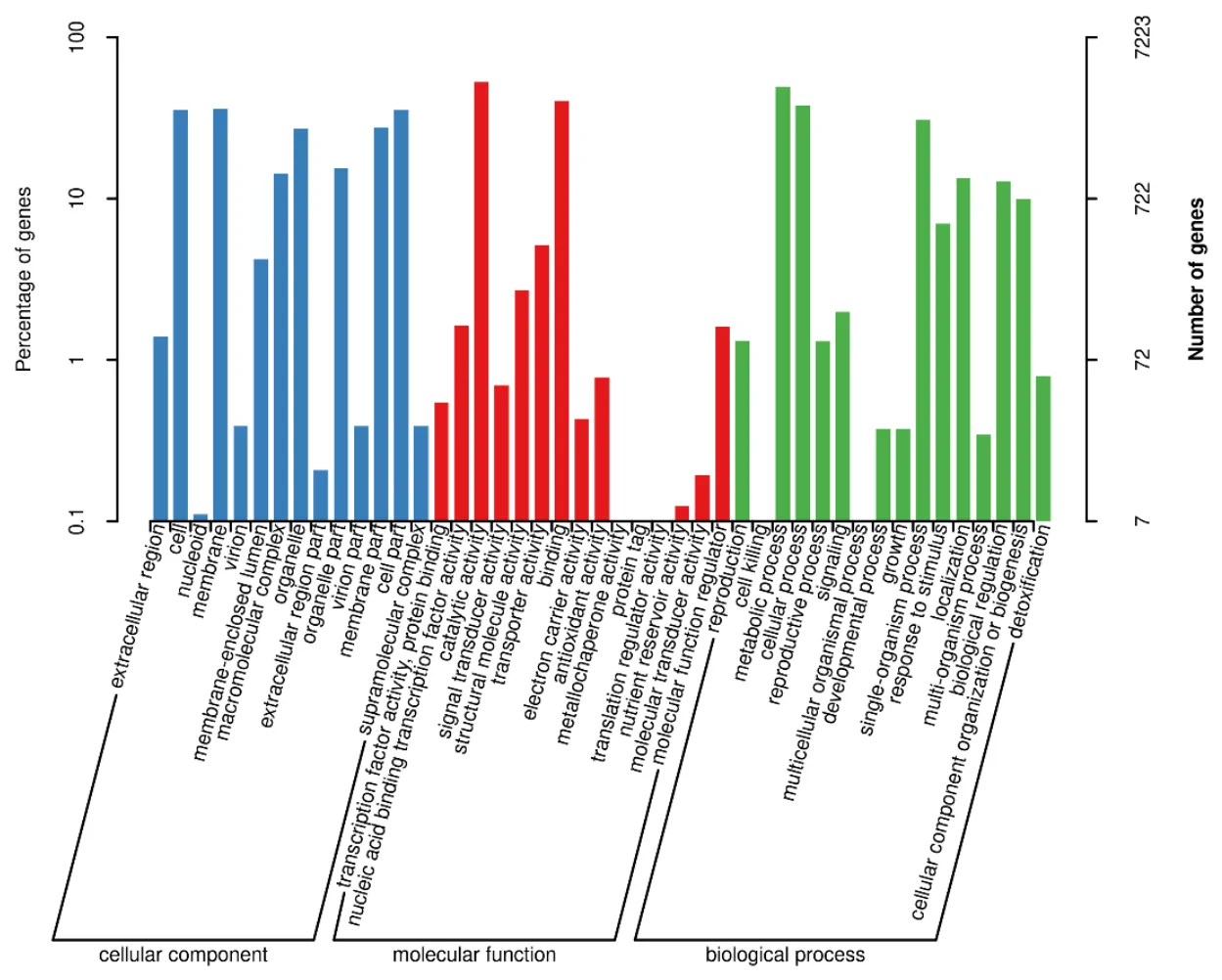
Statistical classification of GO annotations in *P. mesosporus*.

A total of 7223 genes were annotated in the GO database, with an annotation rate of 59.73%. The results of GO functional classification are shown in Figure 6 and are classified into three major categories: Cellular Component, Molecular Function, and Biological Process.

**Figure 7:**
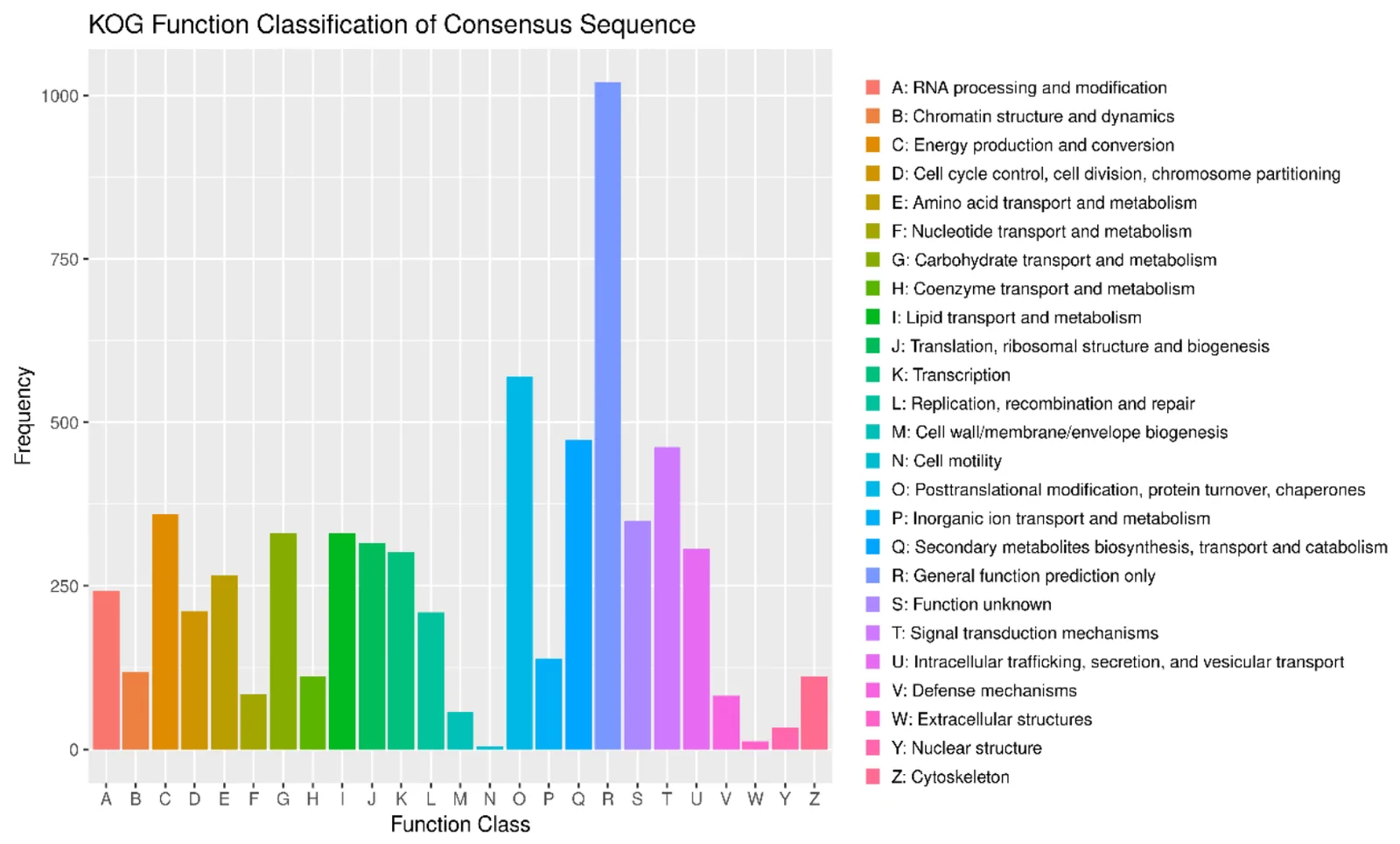
Statistical classification of KOG functional categories in *P. mesosporus*.

The number of annotated genes in the KOG database was 5730, with an annotation rate of 47.39%, as shown in Figure 7. These genes were classified into 25 functional groups.

**Figure 8:**
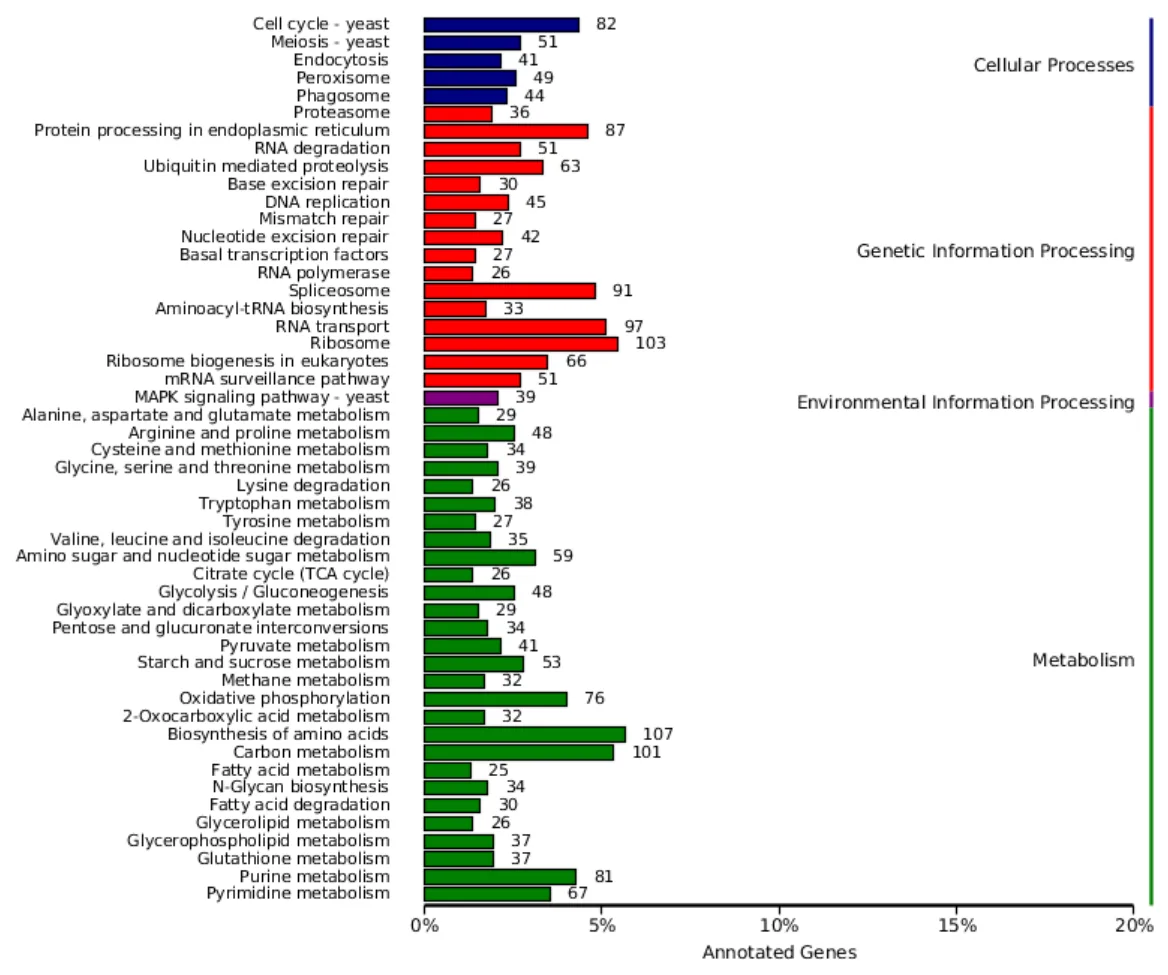
Statistical classification of KEGG annotations in *P. mesosporus*.

A total of 3304 genes were annotated in the KEGG database, with an annotation proportion of 27.32% of the predicted genes, as shown in Figure 8. These genes were divided into four main categories: Cellular Processes (267 genes, 8.08%), Genetic Information Processing (875 genes, 26.48%), Environmental Information Processing (39 genes, 1.18%), and Metabolism (1251 genes, 37.86%), according to KEGG pathway classification.

### 3.5. Mating-Type Genes of P. mesosporus

**Figure 9:**
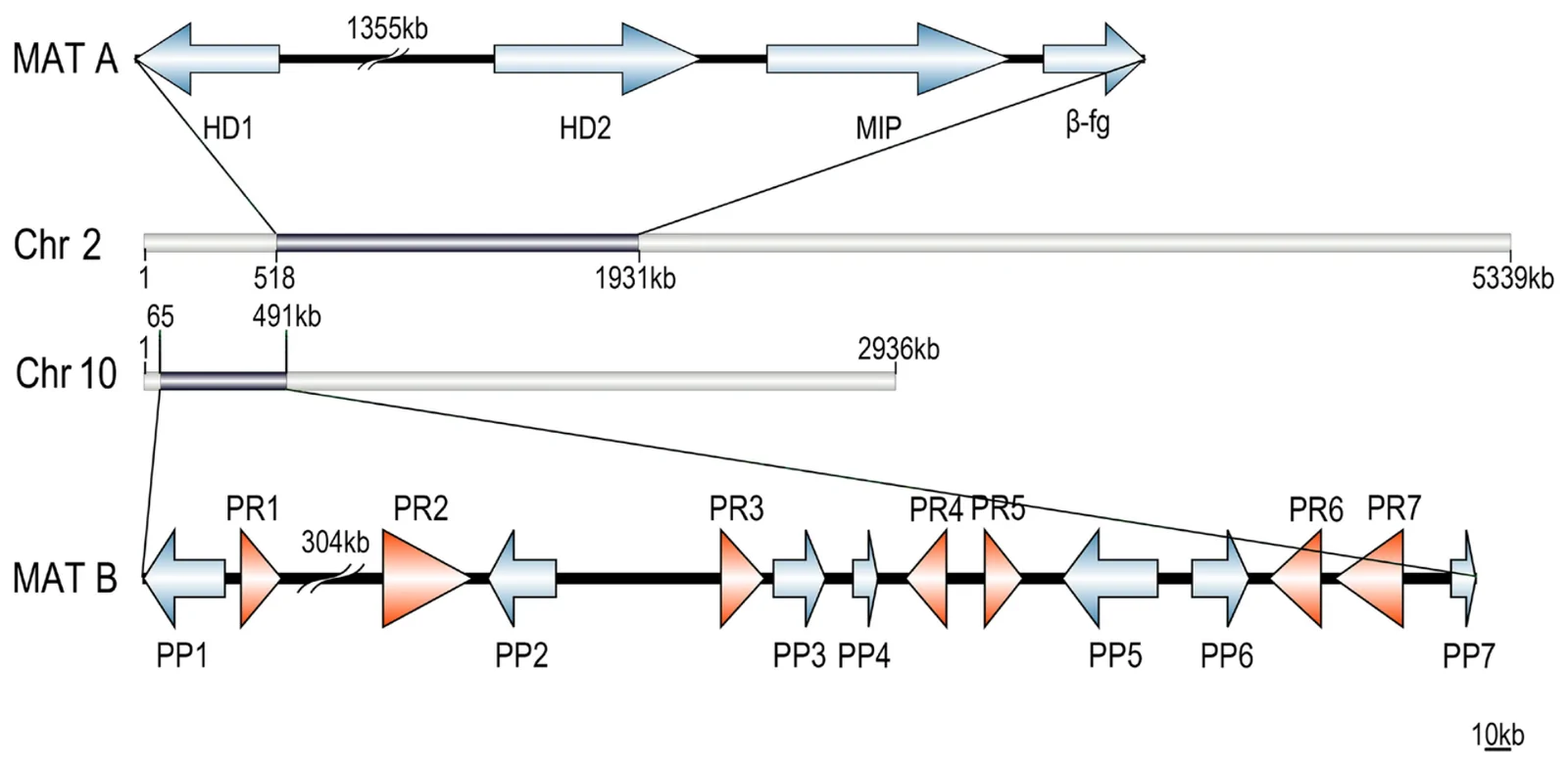
Chromosomal localization and structural diagrams of the mating-type genes MAT A and MAT B in *P. mesosporus*. Chromosomes 2 and 10 are represented by Chr2 and Chr10, respectively.

**Figure 10:**
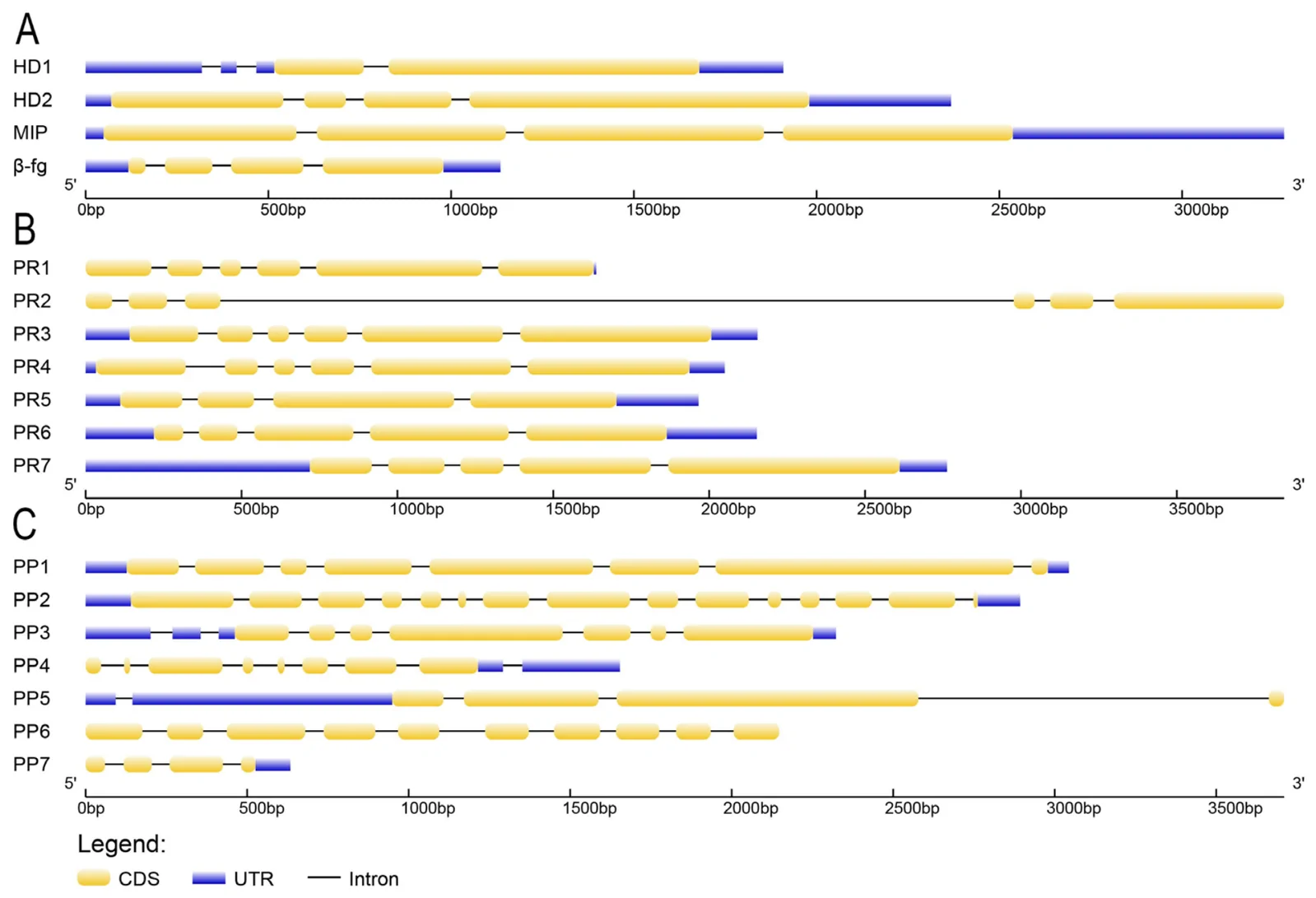
Gene structures of the mating-type genes. (**A**) Genes within the mating-type locus A. (**B**) Pheromone receptors. (**C**) Pheromone precursors. Note: CDS: coding sequence of the protein; UTR: untranslated region. The number of base pairs of each gene is indicated by the scale.

The two mating-type loci of *P. mesosporus*, MAT A and MAT B, were shown by genomic analysis to be located on chromosomes 2 and 10, respectively (Figure 9 and Figure A3). The structure of the mating-type genes is shown in Figure 10. Four genes, *HD1*, *HD2*, *MIP*, and *β-fg*, are contained in the MAT A locus. Seven pheromone receptor genes (*PR1*–*PR7*) and seven pheromone precursor genes (*PP1*–*PP7*) are contained in the MAT B locus. Each gene is composed of a CDS (coding sequence) and UTR (untranslated region). Gene length and relative position in base pairs (bp) are indicated by the scale in the figure. The A and B mating-type loci are located at two unlinked loci in the tetrapolar heterothallic mating system of basidiomycetes, and the formation and stability of the heterokaryon during mating are regulated by them [54]. *P. mesosporus* is confirmed by these results to exhibit typical tetrapolar heterothallic mating characteristics.

#### 3.5.1. Structure and Composition of the Mating-Type Locus A

The mating-type locus A (MAT A) in the *P. mesosporus* genome was identified based on the whole-genome annotation results and conserved domain analysis of the proteins. This locus is composed of four genes: two homeodomain transcription factor genes, *HD1* (EVM01G001020.1) and *HD2* (EVM01G005620.1); a mitochondrial intermediate peptidase *MIP* (EVM01G005760.1); and a β-fold glycoprotein gene *β-fg* (EVM01G005810.1) (Table A3).

**Figure 11:**
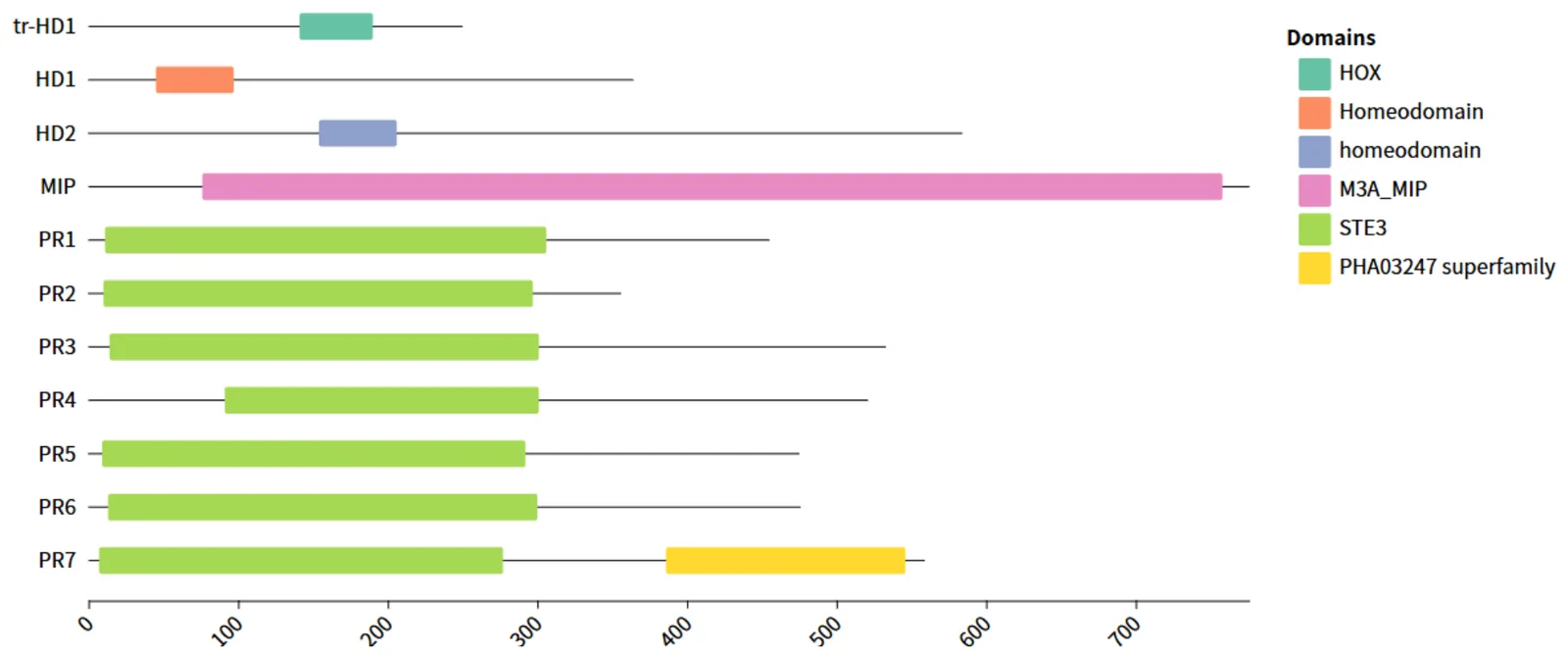
Conserved domains of the mating-type genes.

**Figure A1:**
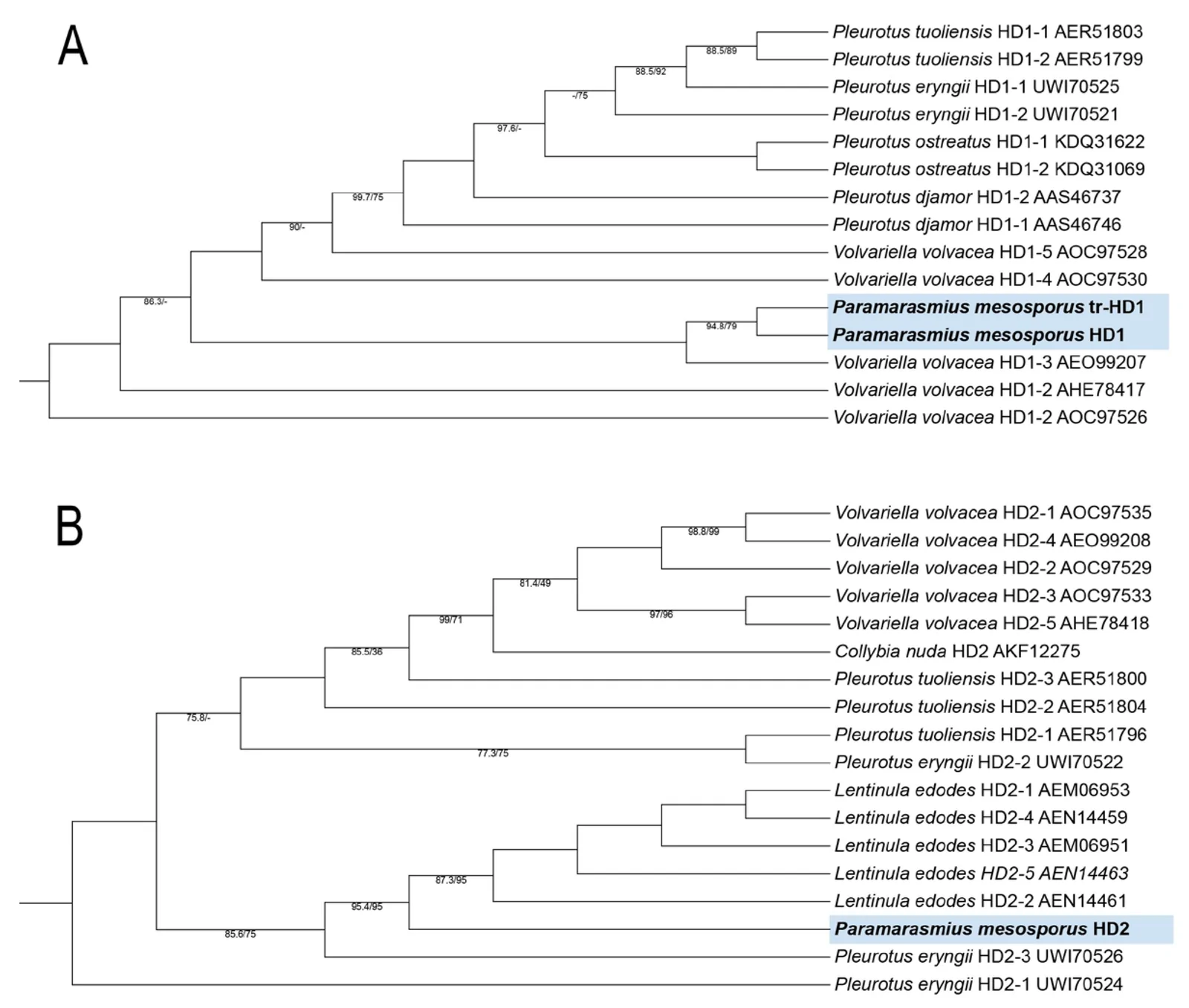
Maximum likelihood phylogenetic trees. (**A**) Homeodomain protein 1 (HD1). (**B**) Homeodomain protein 2 (HD2). The SH-aLRT support values and UFBoot support values from the maximum likelihood analysis are indicated by the numbers below/before and after the branch nodes, respectively.

Phylogenetic analysis was further performed to distinguish the functional status of the HD genes. The presence of three HD homologs in *P. mesosporus*, namely *HD1*, *HD2*, and one functionally degenerated truncated tr-HD gene, was revealed by the results (Figure A1). *HD1* and *HD2* were thereby confirmed to be the functional core of the MAT A locus. Both HD1 and HD2 were shown by protein domain analysis to contain a typical homeodomain (Pfam: PF00046; CDD: cd00086), located at amino acid residues 45–97 and 154–206, respectively, each with a length of 53 amino acids (Figure 11). Notably, the open reading frames of *HD1* and *HD2* are arranged in opposite orientations, which is consistent with the classic structure of the MAT A locus in Basidiomycota. Nuclear localization signal (NLS) prediction for the HD1 and HD2 protein sequences was performed using the PSORT II Prediction website, and HD1 was found to contain three NLS motifs, while HD2 was found to contain ten NLS motifs, suggesting that HD2 may play a more dominant role in nuclear transcriptional regulation. In summary, *HD1*, *HD2*, *MIP*, and *β-fg* are arranged in sequence and together constitute the MAT A locus of *P. mesosporus*.

**Figure 12:**
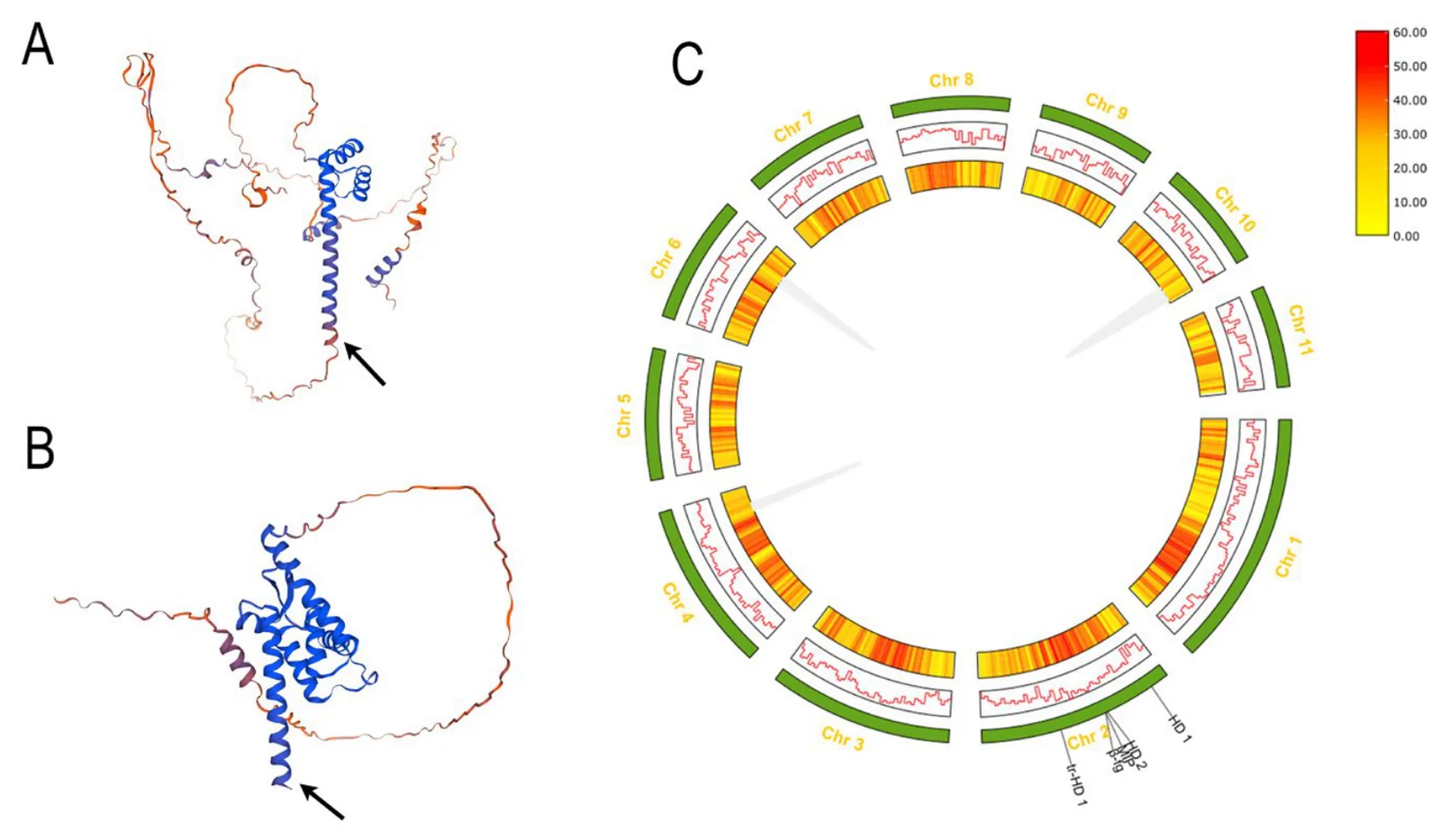
(**A**) Predicted full-length structure of the HD1 protein. (**B**) Predicted full-length structure of the tr-HD1 protein. (**C**) Synteny analysis of *P. mesosporus*. Note: Helix 3 is indicated by the arrows in panels (**A**,**B**).

A third HD gene candidate, *tr-HD1* (EVM01G010840.1), was revealed by genome annotation at the MAT A locus of *P. mesosporus*. Domain degeneration was found to have occurred in this gene. Only the HOX domain (amino acids 141–190, 49 amino acids) is retained by *tr-HD1*, which is four amino acids shorter than the homeodomain of functional *HD1* (45–97 aa, 53 amino acids). Moreover, the domain type was found to have degenerated from a homeodomain to a HOX subtype (Figure 11). DNA-binding and nuclear localization abilities were shown to be lost. Both the DNA-binding site and nuclear localization signals (NLSs) are lacking in *tr-HD1*, thereby rendering it unable to perform the nuclear import function of a transcription factor. Disruption of helix 3 in its three-dimensional protein structure was predicted using SWISS-MODEL homology modeling. leading to a spatial conformational change in the DNA recognition helix (Figure 12). In the context of genome evolution, *tr-HD1* and *HD1* are symmetrically distributed around *HD2*, *MIP*, and *β-fg*. Large-scale rearrangements or duplication events on chromosome 2 were excluded by synteny analysis, and pseudogenization after gene duplication, rather than genomic structural variation, was suggested as the likely origin of *tr-HD1*. Pseudogenization of this gene was indicated by multi-dimensional functional analyses.

#### 3.5.2. Structure and Composition of the Mating-Type Locus B

**Figure 13:**
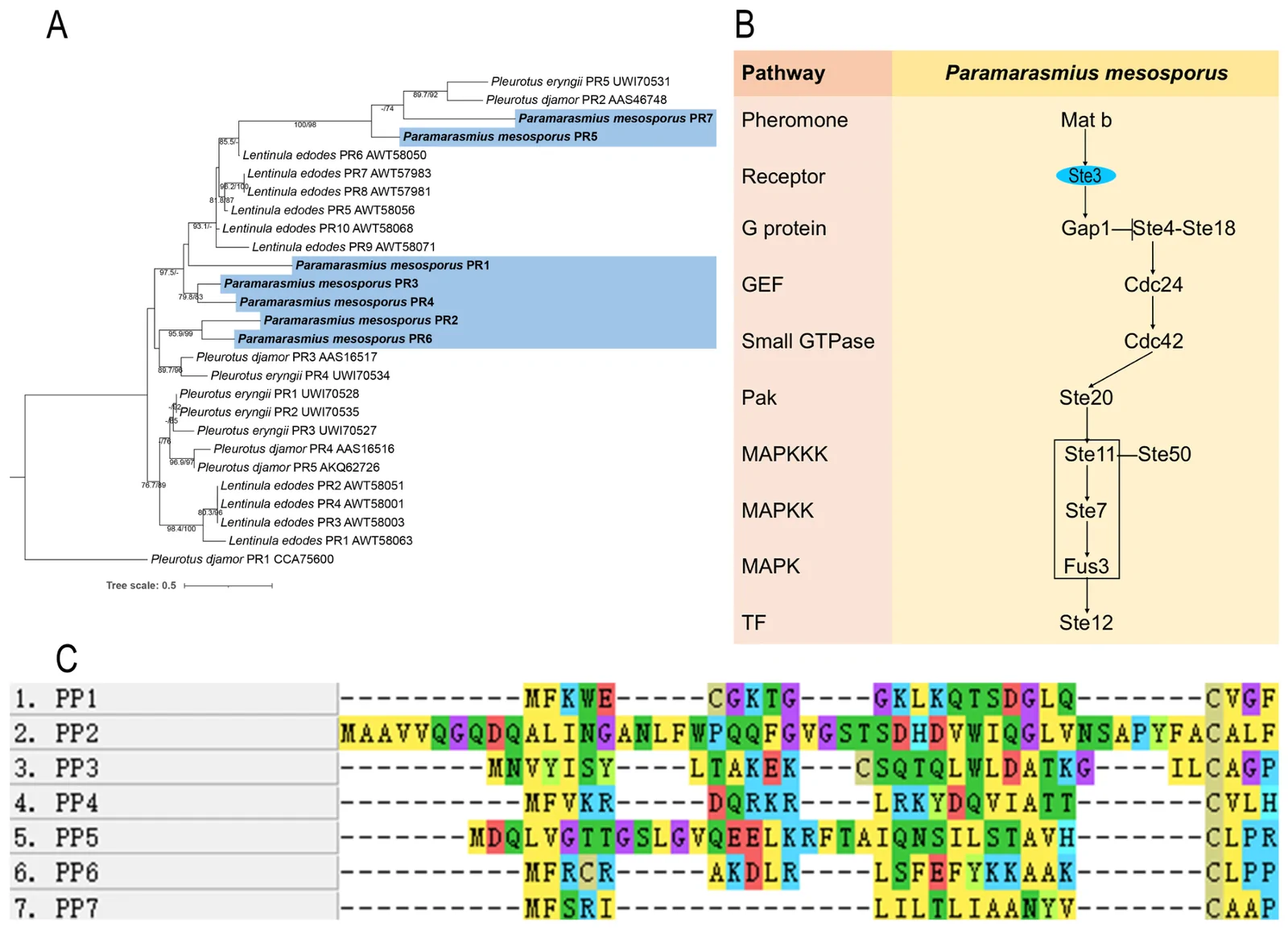
The *PR-PP* system and MAPK signaling pathway at the MAT B locus of *P. mesosporus*. (**A**) Maximum likelihood phylogenetic tree of pheromone receptors *PR1*–*PR7*. (**B**) Genomically predicted model of the MAPK signaling pathway in *P. mesosporus*. (**C**) Alignment of the CAAX motifs of pheromone precursors *PP1*–*PP7*.

**Figure A2:**
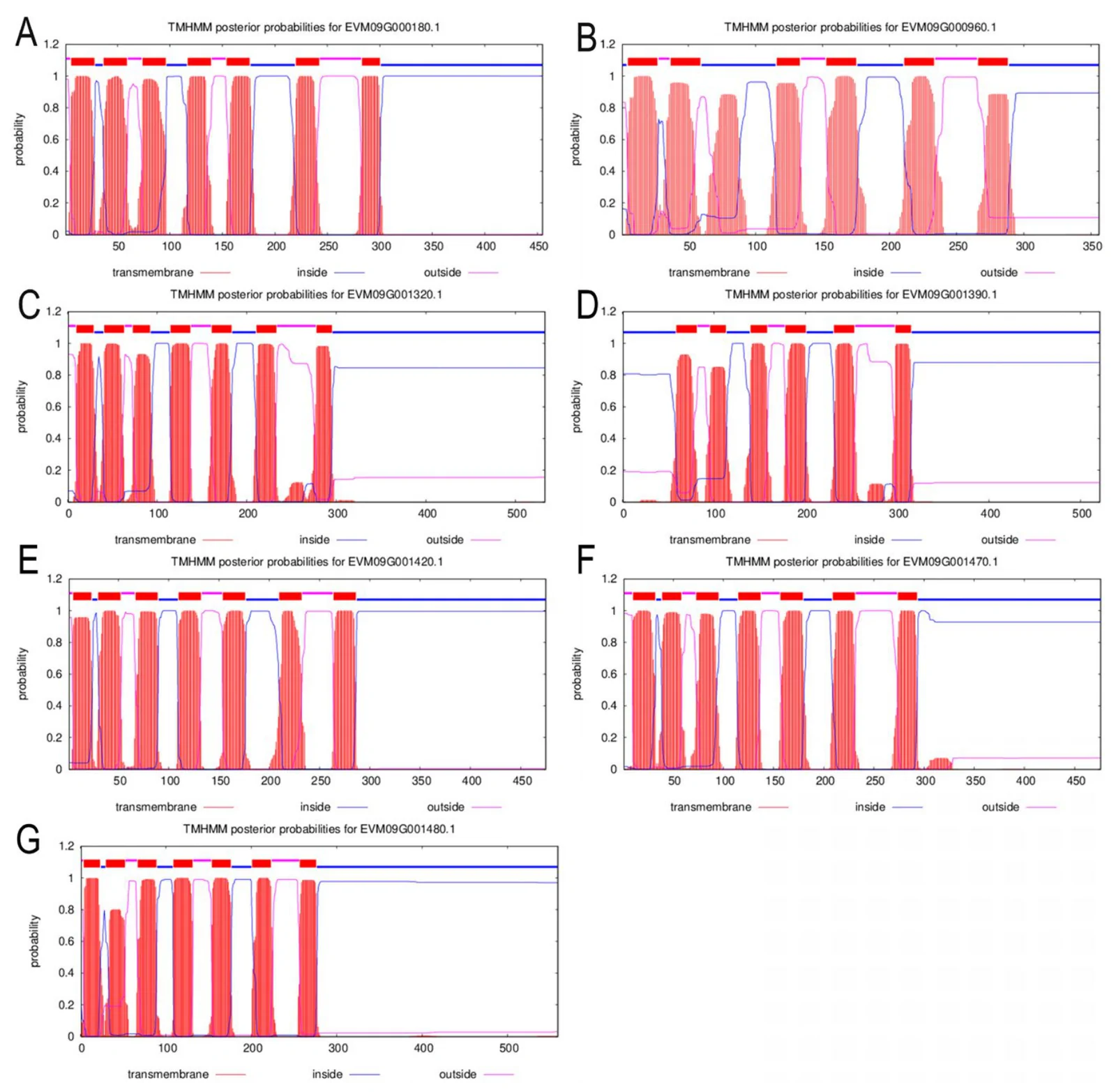
Schematic diagram of the transmembrane structure of pheromone receptors. Note: (**A**–**G**) represent *PR1*–*PR7*, respectively.

**Figure A3:**
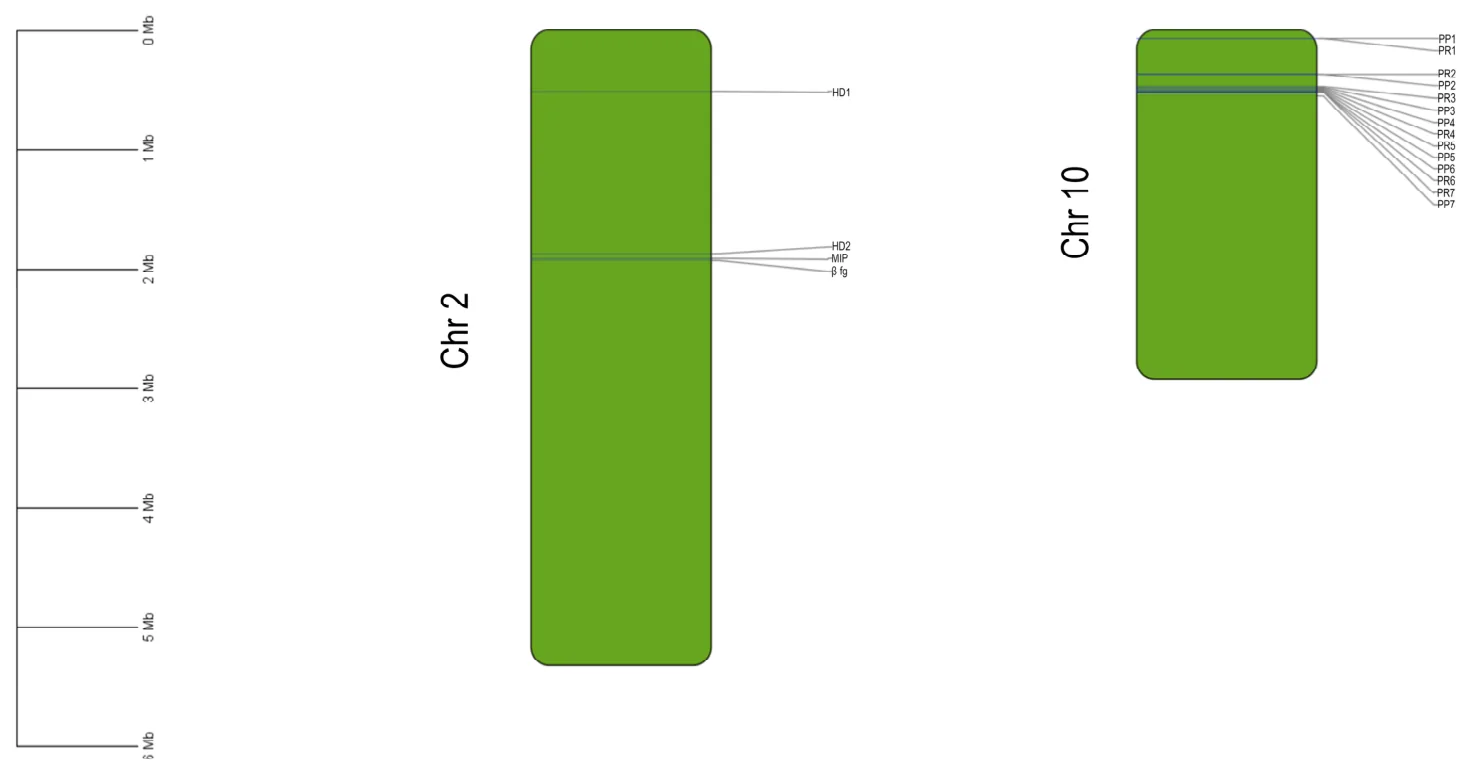
Chromosomal localization of mating-type genes.

Highly expanded features were exhibited by the MAT B locus of *P. mesosporus*, which contains seven pairs of pheromone receptor-precursor (*PR-PP*) genes: *PR1*–*PR7* (EVM09G000180.1, EVM09G000960.1, EVM09G001320.1, EVM09G001390.1, EVM09G001420.1, EVM09G001470.1, EVM09G001480.1) and *PP1*–*PP7* (EVM09G000170.1, EVM09G000980.1, EVM09G001330.1, EVM09G001370.1, EVM09G001440.1, EVM09G001460.1, EVM09G001530.1). *P. mesosporus* is thus recognized as one of the most complex species in terms of the mating-type system among basidiomycetes (Figure 13, Table A4). Structural differentiation was exhibited by the pheromone receptors (*PRs*). G protein-coupled receptors (GPCRs) are encoded by all seven *PRs* genes, and the presence of the STE3 domain (pfam02076) was confirmed by CDD alignment. Functional divergence was revealed by transmembrane structure prediction across the seven receptors. A typical seven-transmembrane structure (N-terminus outside, C-terminus inside) is maintained by *PR1*/3/5/6/7 [55,56], which is consistent with the topological features of functional pheromone receptors. In contrast, only six transmembrane helices are possessed by *PR2* and *PR4*, with the seventh transmembrane helix lacking in them, and these may represent functionally degenerate or subfunctionalized receptors (Figure A2).

Interspecific conservation is lacking in pheromone precursor (*PPs*) gene and protein sequences, making them unsuitable for BLAST homology alignment. A dual-criteria approach was adopted using CAAX motif scanning combined with genomic positional proximity. Seven *PPs* genes were successfully identified within a 5 kb range upstream and downstream of the *PRs* genes by using the conserved CAAX motif (C = cysteine; A = aliphatic amino acid; X = variable residue) as a sequence anchor [56,57] (Figure 13).

#### 3.5.3. Analysis of the Mating-Type MAPK Signaling Pathway

An important role in the hyphal fusion process is played by the MAPK signaling pathway during sexual reproduction of *P. mesosporus*. The core components of the MAPK signaling pathway were systematically mined from the *P. mesosporus* genome based on the dissection of the MAT A and MAT B loci, and the transmission mechanism of pheromone signals from membrane receptors to nuclear transcriptional regulation was thereby elucidated.

The core MAPK signaling components were identified and a pathway model was preliminarily constructed from the *P. mesosporus* genome, based on the seven *PR-PP* pairs at the MAT B locus. These include the heterotrimeric G protein subunits (Ste4/Ste18), the guanine nucleotide exchange factor (Cdc24), the small G protein (Cdc42), the PAK kinase (Ste20), the three-tiered MAPKKK-MAPKK-MAPK kinase cascade module (Ste11-Ste7-Fus3), and the transcription factor (Ste12). Pheromones from different mating-type specificities bind to pheromone receptors (Ste3) [58], activating the receptor and initiating downstream signal transmission. The heterotrimeric G protein is further activated by the activated receptor, resulting in the dissociation of the Ste4 and Ste18 complex and signal transduction. The Cdc24-Cdc42 module is then activated, which further recruits genes is regulated by this model, and the and activates the Ste20 kinase, and the MAPK cascade reaction is thereby initiated [11,58,59]. Ultimately, the signal converges at the transcription factor Ste12 (Figure 13). A mating signal transduction model for this species characterized by “multiple receptor inputs, shared pathway integration, and unified nuclear response” was preliminarily constructed based on these findings (Figure 13). The expression of downstream mating-related cellular response processes mediated by the pheromone signals from the mating-type B locus is completed through it.

## 4. Discussion

The biological characteristics of edible mushroom are closely linked to their genetic background. *P. mesosporus* completes the cycle from spawning to fruiting in approximately 7 days; this trait is currently shared only by *Volvariella volvacea* [60]. Typical characteristics of thermophilic straw-decay fungi have been demonstrated for this species by domestication and fruiting experiments: the optimal fruiting temperature ranges from 32 to 36 °C, with fruiting body development being inhibited below 28 °C; moreover, a significant preference for wheat straw substrate has been observed as evidenced by markedly improved mycelial growth on wheat straw-amended media. Combined with the observation that wild strains predominantly occur in summer on the roots of *Imperata cylindrica*, *P. mesosporus* is inferred to be a thermophilic straw-decay fungus. At present, commercially cultivated edible and medicinal fungal species are predominantly mesophilic to psychrophilic species, with only a very limited number of thermophilic cultivars such as *V. volvacea* [60], *Phlebopus portentosus* [61], *Buchwaldoboletus xylophilus* [62], and *Pleurotus giganteus* [63]. Considerable potential for commercial development is possessed by *P. mesosporus* as a delicious thermophilic straw-decay fungus. Collectively, *P. mesosporus* is characterized by thermotolerance, rapid growth, and a marked preference for straw-based substrates such as wheat straw, features that are well aligned with cultivation demands in the context of global warming. However, industrial application is limited by its low yield. The genetic limitations imposed by habitat adaptation cannot be overcome by traditional domestication approaches, and the integration of modern molecular breeding techniques is urgently required for the development of high-yield, high-quality elite cultivars.

**Figure 14:**
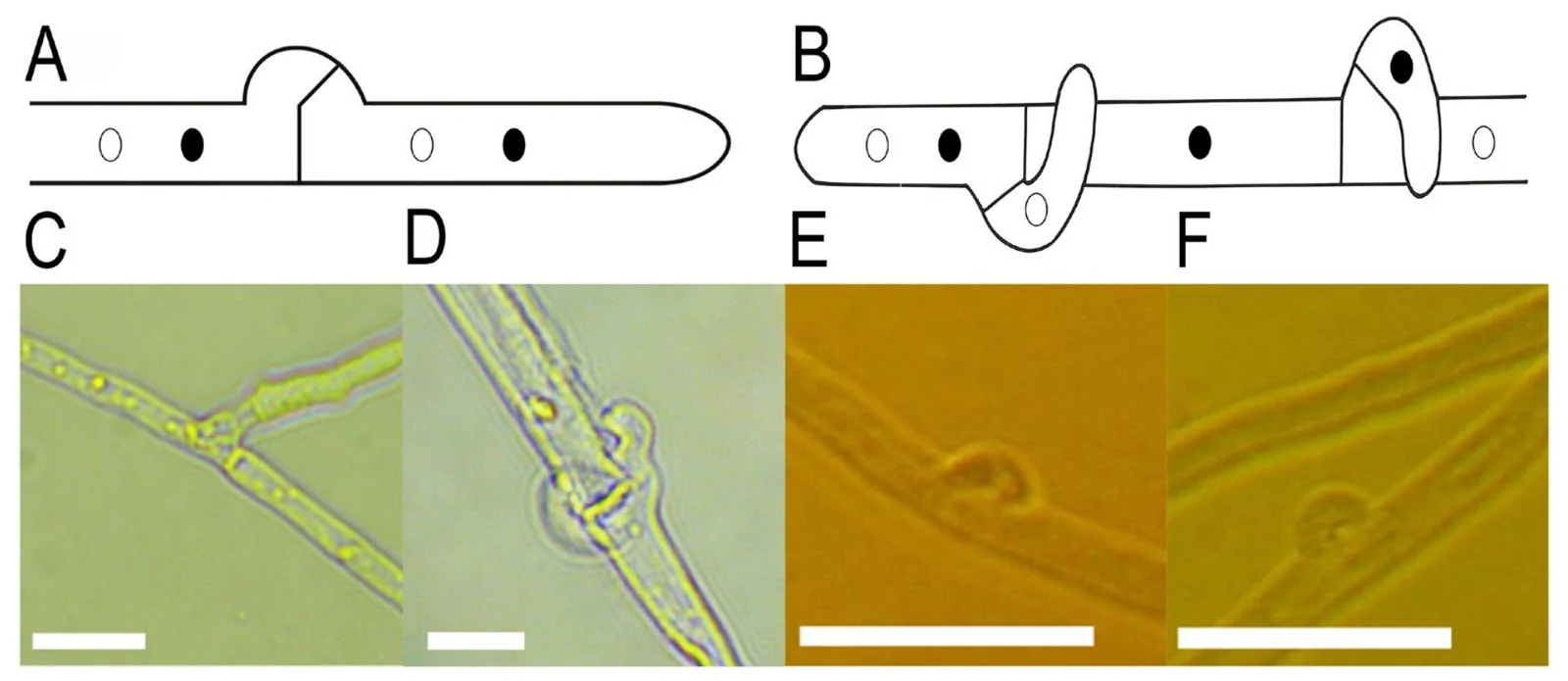
(**A**) Schematic diagram of a true clamp connection. (**B**) Schematic diagram of a pseudo-clamp connection. (**C**) Monokaryotic hyphae of *P. mesosporus*. (**D**) Dikaryotic hyphae of *P. mesosporus*. (**E**,**F**) Pseudo-clamp connections. Note: Scale bars = 10 μm. (**A**,**B**) were drawn based on studies by Kothe et al. [67] and Yan et al. [68]; (**E**,**F**) are cited from Boontawon et al. [64].

Traditional mating-type identification in edible mushroom has been carried out using a workflow involving protoplast monokaryotization, monospore hybridization, and nuclear migration tests. However, this approach is considered labor-intensive, time-consuming, and susceptible to misjudgment, which is caused by pseudo-clamp connections in tetrapolar systems [25,64] (Figure 14). In this study, whole-genome sequencing was employed, and it was revealed that MAT A is located on chromosome 2 and Mat B on chromosome 10, with the two mating-type loci being distributed in a nunlinked manner. A tetrapolar heterothallic mating system is governed by two unlinked mating-type loci, A and B, located on different chromosomes [65,66]. Therefore, the tetrapolar heterothallic mating system of *P. mesosporus* was confirmed at the chromosomal level. This method is characterized by accurate and stable results, with phenotypic interference being effectively avoided [25], thereby laying a solid foundation for the rapid identification of mating types in subsequent large-scale breeding materials.

**Figure 15:**
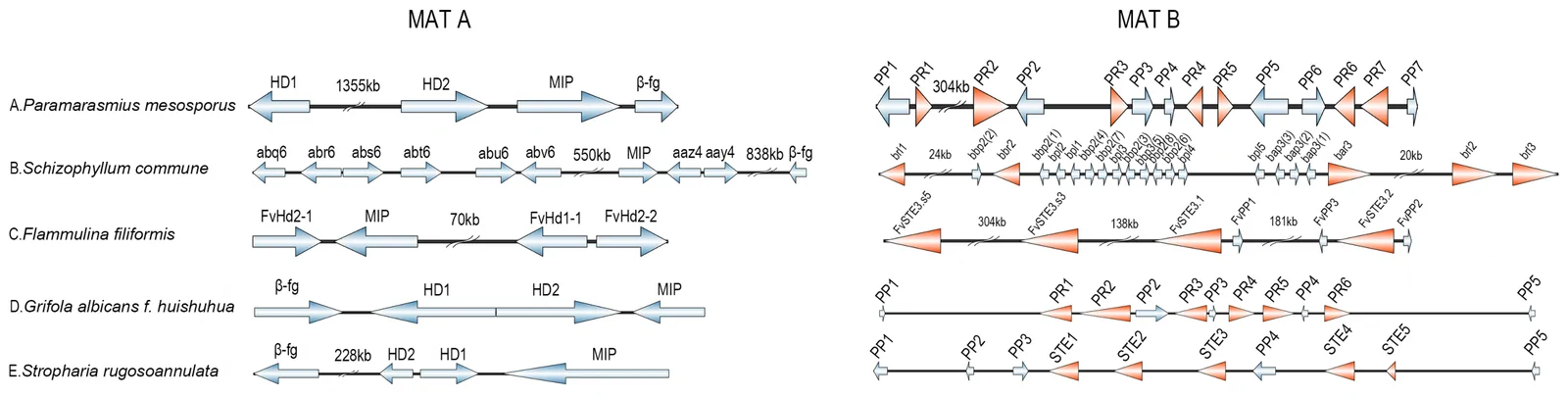
Gene structure of mating-type loci in *P. mesosporus*, *Schizophyllum commune*, *Flammulina filiformis*, *Grifola albicans f. huishuhua*, and *Stropharia rugosoannulata*. Panels B to E were drawn according to the studies of Bao et al. [71], Zhang et al. [25], and Zhang et al. [70], respectively.

The tight linkage between *HD1* and *HD2* is a typical feature of the MAT A locus, through which the activation of diploid-specific transcriptional programs is induced by MAT A via heterodimerization [54,69]. Although functionally active *HD1* and *HD2* were identified in *P. mesosporus*, their physical distance is 1355 kb. This is extremely rare among known basidiomycetes (Figure 15). Similar loose structures have also been observed in *Flammulina filiformis*, *Schizophyllum commune*, and *Stropharia rugosoannulata* [70,71]. However, more drastic chromosomal rearrangements of the A locus are suggested by the extreme spacing in *P. mesosporus*. The region between *HD1* and *HD2* is highly divergent, and recent rearrangement events are ruled out indicating that this structure is represented as a long-term evolutionarily stable feature. Space for sequence translocation and gene duplication may be provided by the wide intergenic distance [70,72]. This finding is consistent with the presence of *tr-HD1* and pseudogenization tracks, and the “loose architecture” of the MAT A locus is suggested to serve as the structural basis for its evolutionary plasticity.

Ten NLSs are contained in *HD2*, which is significantly more than in *HD1* (three). A more dominant role in nuclear regulation is suggested to be assumed by *HD2*, considering that *HD2* is tightly linked with *MIP* and *β-fg* as a core gene cluster, whereas *HD1* is isolated 1355 kb apart. Stable nuclear localization and efficient nuclear import are likely ensured by the higher density of NLSs [73,74], and the spatiotemporal regulatory delays caused by the distant translocation of *HD1* are thereby compensated for. An evolutionary adaptive strategy that maintains precise mating regulation within the loosely organized MAT A locus may be represented by this “core-periphery” spatial-functional differentiation. Despite the extreme expansion of the *HD1*-*HD2* distance, the core conserved module of the basidiomycete MAT A locus is still retained by *P. mesosporus*, comprising an intact *MIP*-*β-fg*-*HD2* gene cluster [54]. A balancing mechanism in fungal mating-type loci between functional constraint and evolutionary innovation is revealed by this combined pattern of “conserved core plus variable architecture”. The core regulatory module remains indispensable, whereas pronounced lineage-specific plasticity is exhibited by the overall locus organization.

A trajectory of “innovation-burst-simplification” is followed by fungal genome evolution, with simplification dominating in the long term and being interspersed with episodes of complexification [75]. Suppressed recombination is experienced by mating-type loci, which functions in species recognition. Reduced selection efficiency and accumulation of genetic burden are caused by this, and hotspots for gene degeneration are thereby created [76]. Following whole-genome duplication, the rate of gene loss accelerates substantially [77]. A typical manifestation of this principle is the pseudogenization of *tr-HD1* in *P. mesosporus*. Nuclear localization is blocked by the loss of NLSs, and DNA-binding ability is impaired by the disruption of helix 3 [71]. Once function is lost, expression decays to silencing, and the gene eventually tends to be completely eliminated from the genome. Notably, *tr-HD1* and *HD1* are symmetrically arranged and equidistant from the core gene cluster (*HD2*-*MIP*-*β-fg*), and a functional backup system that they once constituted is suggested. Under the pressure of mutation accumulation caused by recombination suppression, the immediate impact of deleterious mutations on mating function could be buffered by such a backup mechanism, and evolutionary robustness to the MAT A locus was thereby conferred. As *HD1* became functionally stable, *tr-HD1* shifted from a backup to a redundant element, and its degeneration represents an inevitable outcome of the MAT A locus trending toward streamlining and efficiency.

**Figure A4:**
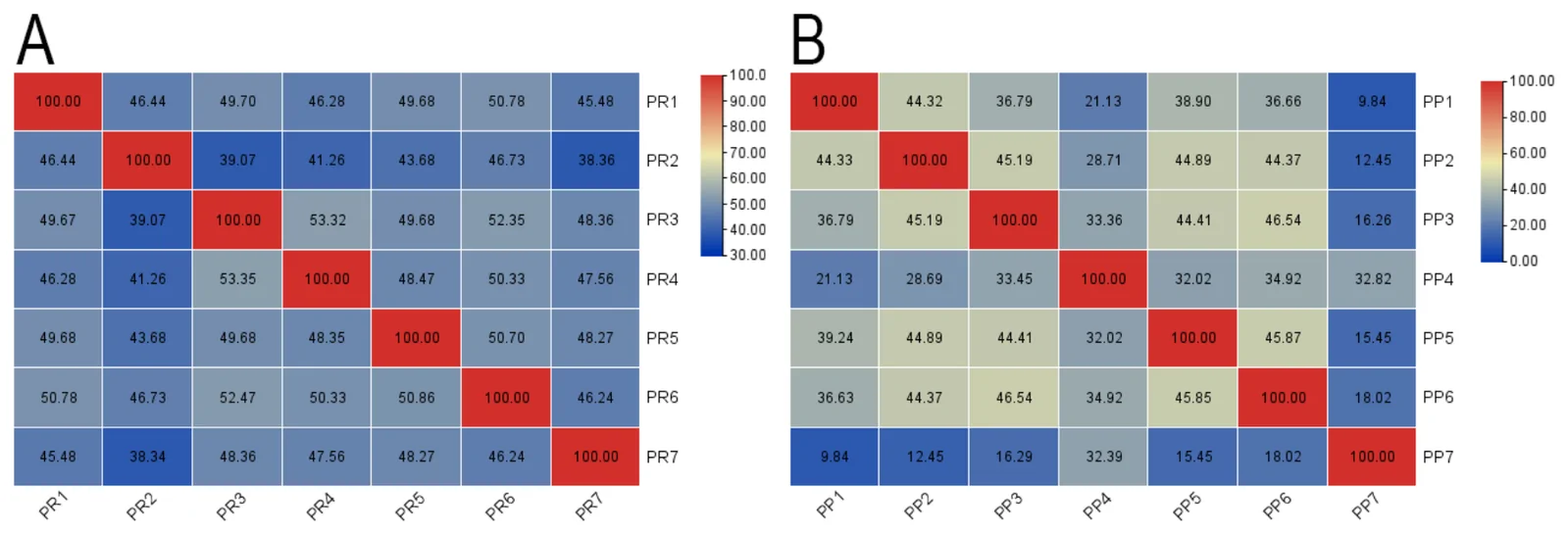
Heatmap of similarity matrix based on CDS sequences. (**A**) Pheromone receptors. (**B**) Pheromone precursors.

The mating-type system of *P. mesosporus* is characterized by asymmetric evolution between the A and B loci. A streamlining process of “backup → degeneration → deletion” was undergone by the MAT A locus, whereas an evolutionary paradox was formed by the Mat B locus in contrast to the streamlining of MAT A, with a complex system of seven pairs of pheromone receptors (PR) and pheromone precursors (PP) being maintained through “recruitment-amplification-divergence”. The regulation of nuclear exchange, septal dissolution, and clamp connection formation is regulated by the pheromone receptor and pheromone precursor system [11,78,79]. Fungal pheromone receptors typically contain seven transmembrane domains. However, variation in the number of receptors and transmembrane domains is observed among different species [79]. All seven *PRs* in this species were found to possess six to seven transmembrane structures (Figure A2)**,** with a tightly linked gene cluster being formed by *PR3*-*PR7*, while relatively distant positions (304 kb/104 kb) were occupied by *PR1*/*PR2*. Significant divergence and ancient origin of PR genes were revealed by genome alignment [80], and these genes were ruled out as products of recent tandem duplication (Figure A4). Seven pairs of *PR-PP* genes are distributed in a clustered manner at the Mat B locus, constituting a complex interaction network of one locus with multiple recognition pairs. Although more flexible mating recognition ability is conferred on the species by this amplification pattern, the complexity of parental pairing in crossbreeding programs is also increased by it. Notably, a conserved CaaX motif (protein length 20–52 aa) is contained by all seven pheromone precursors, ensuring the basic functions of post-translational modification and membrane anchoring. However, unlike most basidiomycetes, significant divergence was observed in the upstream AF/EH/EA recognition motifs in *P. mesosporus* [25,81]. Only the core CaaX motif of pheromone precursors was found to be highly conserved, with significant sequence diversity and divergence being exhibited by the upstream characteristic small motifs (Figure 13 and Figure A4). Strong constraint on the core functional element but rapid divergence in recognition regions is featured by this evolutionary pattern. The core function of signaling molecules is not only locked by such a strategy, but evolutionary flexibility for the mating recognition system is also preserved, echoing the “sub-locus specific recognition” mechanism reported in *Lentinula edodes* by Kim et al. [82]. In this way. The core function of signaling molecules is not only preserved but also “flexible space” is provided for the mating recognition system by this strategy, enabling flexible adjustment with environmental changes and avoiding hybridization between different populations. Consequently, the genetic burden is reduced by the streamlined A locus, whereas the recognition precision is enhanced by the complexity of the B locus, carrying dual significance for molecular breeding.

A preliminary MAPK signaling pathway model (Ste3 → G protein → Cdc24/42 → Ste20 → MAPK cascade → Ste12) at the MAT B locus was preliminarily constructed based on genome annotation in this study, providing a new perspective for the elucidation of the mating regulatory network of *P. mesosporus*. The MAPK pathway may be activated in a combinatorial manner by the seven pairs of *PR-PP* in this species to achieve refined mating-type discrimination, as hypothesized based on the “single pheromone-multi-receptor sublocus recognition” mechanism revealed by Kim et al. [82]. However, this pathway currently represents a genome-predicted model, and several critical issues remain to be clarified by gene functional validation (e.g., heterologous expression in yeast, CRISPR-mediated knockout) and protein interaction analysis (e.g., Co-IP, BiFC), including whether differential signal transduction efficiency is caused by the six-transmembrane structure of *PR2*/*PR4*, whether competition for shared downstream components is exhibited by different *PR-PP* pairs, and how clamp connection formation is regulated by Ste12 target genes. Ultimately, an early molecular diagnostic system for hybrid compatibility in *P. mesosporus* encompassing “genomic analysis → marker development → precise pairing → phenotypic validation” is to be established, along with a molecular design breeding technology system, thereby promoting the transformation of *P. mesosporus* from wild resources to cultivated new cultivars with high yield, superior quality, and thermotolerance.

## 5. Conclusions

The first successful domestication of *Paramarasmius mesosporus* is reported under artificial conditions in this study. A chromosome-level genome fine map (46.57 Mb, 11 chromosomes) of this species was also obtained. The molecular basis of its tetrapolar heterothallic mating system was systematically dissected. The MAT A locus (chromosome 2) is composed of a functional *HD1*-*HD2* pair and a tightly linked *MIP*-*β-fg* gene cluster. The physical distance between *HD1* and *HD2* is 1355 kb. Additionally, a *tr-HD1* pseudogene was identified, which appears to be on a “backup-to-degeneration” trajectory. Seven *PR-PP* pairs of ancient origin are contained in the MAT B locus (chromosome 10). An evolutionary pattern of “CaaX core conservation versus recognition region diversification” is exhibited by the pheromone precursors. Moreover, transmembrane domain variations were observed in *PR2* and *PR4*. A four-step molecular marker-assisted breeding scheme is proposed based on these findings. A theoretical foundation for breaking the industrial bottlenecks of low yield and insufficient protein content is laid by this work. The transformation of *P. mesosporus* from a wild resource into new varieties with high yield, superior quality, and high-temperature tolerance will be facilitated through design-driven breeding. Ultimately, large-scale cultivation and industrial utilization of this species will be promoted by this advancement.

## Data Availability

The ITS sequences obtained in this study have been submitted to the GenBank database, accession numbers: PZ506055 (M20250718), PZ506056 (Z20250718), PZ506057 (Y20250718).

## Appendix A

**Table A1:** Hi-C assembly data statistical table.

Group	Sequence Number	Sequence Length (bp)
Chr 1	4	6,935,470
Chr 2	1	5,339,614
Chr 3	1	4,981,969
Chr 4	1	4,603,359
Chr 5	1	3,560,520
Chr 6	1	3,475,424
Chr 7	1	3,369,991
Chr 8	1	3,211,266
Chr 9	1	3,154,206
Chr 10	1	2,936,468
Chr 11	1	2,763,944
Total Sequences Clustered (Ratio %)	14 (16.47)	44,332,231 (95.18)
Total Sequences Ordered and Oriented (Ratio %)	14 (100.00)	44,332,231 (100.00)
Total unanchored sequences (Ratio %)	71 (83.53)	2,243,477 (4.82)

**Table A2:** Statistics of gene prediction results.

Method	Software	Species	Gene Number
Ab initio based	Augustus		10,793
	Genscan		6548
	GeneID		12,960
	GlimmerHMM		13,078
	SNAP		12,557
RNA-seq based	PASA v2.0.2		19,561
	TransDecoder v2.0		25,114
Homology based	GeMoMa	*Marasmius fiardii*	10,659
		*Paramarasmius palmivorus*	11,497
		*Marasmius oreades*	9681
Integration	EVM v1.1.1		12,599

**Table A3:** Mating-type locus A.

Gene ID	Exon	Intron	Chr2 Location	CDS	Amino Acid Number	Direction of Sequence
*HD1* (EVM01G001020.1)	4	3	518,013–519,921	1095	365	−
*HD2* (EVM01G005620.1)	4	3	1,875,146–1,877,513	1755	584	+
*MIP* (EVM01G005760.1)	4	3	1,917,816–1,921,094	2331	776	+
*β-fg* (EVM01G005810.1)	4	3	1,929,988–1,931,122	705	234	+

Note: +, positive strand; −, negative strand.

**Table A4:** Mating-type locus B.

Gene ID	Exon	Intron	Chr10 Location	CDS	Amino Acid Number	Direction of Sequence
EVM09G000180.1 (*PR1*)	6	5	69,053–70,690	1368	455	+
EVM09G000960.1 (*PR2*)	6	5	366,338–370,181	1027	342	+
EVM09G001320.1 (*PR3*)	6	5	472,376–474,530	1719	532	+
EVM09G001390.1 (*PR4*)	6	5	495,965–498,014	1719	572	−
EVM09G001420.1 (*PR5*)	4	3	507,416–509,381	1644	547	+
EVM09G001470.1 (*PR6*)	5	4	521,913–524,065	1479	492	−
EVM09G001480.1 (*PR7*)	5	4	525,050–527,812	1971	656	−
EVM09G000170.1 (*PP1*)	8	7	65,573–68,616	2823	940	−
EVM09G000980.1 (*PP2*)	15	14	373,013–375,905	2550	849	−
EVM09G001330.1 (*PP3*)	9	8	479,059–481,381	1698	565	+
EVM09G001370.1 (*PP4*)	9	8	491,432–493,085	897	298	+
EVM09G001440.1 (*PP5*)	5	4	511,726–515,435	2430	809	−
EVM09G001460.1 (*PP6*)	10	9	519,906–522,052	1806	601	+
EVM09G001530.1 (*PP7*)	4	3	556,686–557,319	426	141	+

Note: +, positive strand; −, negative strand.

**Table A5:** Transmembrane helical structure of pheromone receptor.

Pheromone Receptor Gene ID	Amount	The Transmembrane Domain of Amino Acids							Probability of N-Terminus on Cytoplasmic Side
		T	T	T	T	T	T	T	
		M1	M2	M3	M4	M5	M6	M7	
EVM09G000180.1 (*PP1*)	7	6	37	74	117	154	220	283	0.02095
		28	59	96	139	176	242	300	
EVM09G000960.1 (*PP2*)	6	5	37	116	153	211	266		0.16561
		27	59	133	175	233	288		
EVM09G001320.1 (*PP3*)	7	10	41	73	115	161	211	278	0.06968
		29	63	92	137	183	233	295	
EVM09G001390.1 (*PP4*)	6	59	96	140	178	231	298		0.80886
		81	113	158	200	253	315		
EVM09G001420.1 (*PP5*)	7	5	30	67	110	154	210	264	0.04142
		23	52	89	132	176	232	286	
EVM09G001470.1 (*PP6*)	7	10	39	73	115	157	209	274	0.01679
		32	58	95	137	179	231	293	
EVM09G001480.1 (*PP7*)	7	4	30	67	109	154	201	257	0.19983
		23	52	89	131	176	223	276	
